# Synthesis and In Vitro Evaluation of 8-Pyridinyl-Substituted Benzo[*e*]imidazo[2,1-*c*][1,2,4]triazines as Phosphodiesterase 2A Inhibitors

**DOI:** 10.3390/molecules24152791

**Published:** 2019-07-31

**Authors:** Rien Ritawidya, Friedrich-Alexander Ludwig, Detlef Briel, Peter Brust, Matthias Scheunemann

**Affiliations:** 1 Department of Neuroradiopharmaceuticals, Institute of Radiopharmaceuticals Cancer Research, Helmholtz-Zentrum Dresden-Rossendorf, 04318 Leipzig, Germany; 2 Center for Radioisotope and Radiopharmaceutical Technology, National Nuclear and Energy Agency (BATAN), Puspiptek Area, Serpong, South Tangerang 15310, Indonesia; 3 Pharmaceutical/Medicinal Chemistry, Institute of Pharmacy, Faculty of Medicine, Leipzig University, Brüderstraße 34, 04103 Leipzig, Germany

**Keywords:** Phosphodiesterase 2A (PDE2A), Positron Emission Tomography (PET), Benzoimidazotriazine (BIT), fluorinated, Mouse Liver Microsomes (MLM)

## Abstract

Phosphodiesterase 2A (PDE2A) is highly expressed in distinct areas of the brain, which are known to be related to neuropsychiatric diseases. The development of suitable PDE2A tracers for Positron Emission Tomography (PET) would permit the in vivo imaging of the PDE2A and evaluation of disease-mediated alterations of its expression. A series of novel fluorinated PDE2A inhibitors on the basis of a Benzoimidazotriazine (BIT) scaffold was prepared leading to a prospective inhibitor for further development of a PDE2A PET imaging agent. BIT derivatives (**BIT1**–**9**) were obtained by a seven-step synthesis route, and their inhibitory potency towards PDE2A and selectivity over other PDEs were evaluated. **BIT1** demonstrated much higher inhibition than other BIT derivatives (82.9% inhibition of PDE2A at 10 nM). **BIT1** displayed an IC_50_ for PDE2A of 3.33 nM with 16-fold selectivity over PDE10A. This finding revealed that a derivative bearing both a 2-fluoro-pyridin-4-yl and 2-chloro-5-methoxy-phenyl unit at the 8- and 1-position, respectively, appeared to be the most potent inhibitor. In vitro studies of **BIT1** using mouse liver microsomes (MLM) disclosed **BIT1** as a suitable ligand for ^18^F-labeling. Nevertheless, future in vivo metabolism studies are required.

## 1. Introduction

Cyclic nucleotide Phosphodiesterases (PDEs) represent a class of enzymes catalyzing the hydrolysis of the intracellular second messengers, cyclic Adenosine Monophosphate (cAMP) and cyclic Guanosine Monophosphate (cGMP) [1]. cAMP and cGMP are involved in a great variety of cellular functions related to physiological and pathophysiological processes in brain and periphery [2,3,4,5,6].

The 11 family members of PDEs are encoded by 21 genes and classified by their substrate specificity [7,8,9,10]. PDEs 4, 7, and 8 are cAMP-specific and PDEs 5, 6, and 9 cGMP-specific, and others (PDEs 1, 2, 3, 10, and 11) hydrolyze both substrates [7,9,11]. 

The dual substrate enzyme PDE2A is highly expressed in some brain areas, such as nucleus accumbens, cortex, hippocampus [7], striatum, amygdala [12], substantia nigra, and olfactory neurons [13,14,15], thus being involved in complex neuronal processes like learning, concentration, memory, emotion, and related diseases [8,9]. The inhibition of PDE2A will increase the intracellular levels of cGMP and cAMP in PDE2A-abundant tissues and may result in improvement of neuroplasticity and memory function [8]. 

The activity of PDE2A inhibitors related to cognitive improvement has been evaluated in animal models [1,7,16,17]. These results suggest the use of PDE2A inhibitors for treatment of neuropsychiatric diseases such as Alzheimer’s disease and schizophrenia [18].

**Figure 1:**
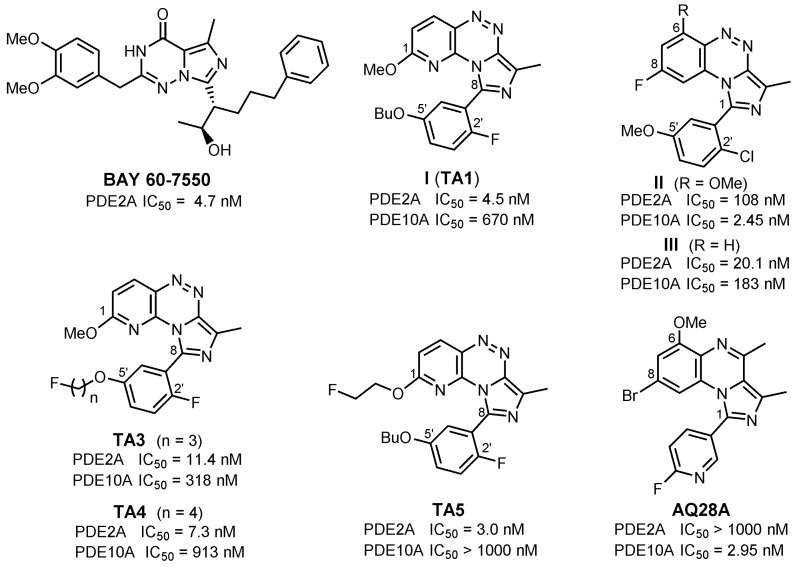
Different PDE inhibitors [15,19,20,21,22,23,24,25,26,27].

Up to now, there have been several PDE2A inhibitors reported. **BAY 60-7550** (Figure 1), the first highly-selective PDE2A inhibitor, has been widely used to evaluate PDE2A activity. However, this compound shows a poor pharmacokinetic profile, as well as poor ability to cross the blood-brain barrier [11,16]. The finding of **BAY 60-7550** triggered many pharmaceutical companies to discover potent PDE2A inhibitors for potential use in treating a variety of brain diseases [11].

In 2010, Biotie Therapies and Wyeth claimed a series of 1,2,4-triazine- and pyrazine-containing tricyclic compounds exhibiting dual inhibition against both PDE2A and PDE10A as therapeutic targets [25,26,27,28]. The triazine series comprises benzo- and pyridine-fused imidazo[5,1-*c*][1,2,4]triazine derivatives [27], some of which are depicted in Figure 1 (Compounds **I** (**TA1**), **II**, and **III**), along with their binding affinities. In 2015, our group reported on the first PDE2A PET tracers on the basis of a Pyridoimidazotriazine (PIT) scaffold starting from **I** (**TA1,** Figure 1), as the lead compound. Two fluoroalkyl derivatives, **TA3** and **TA4** (Figure 1), demonstrated high affinity towards PDE2A with 28-fold and 125-fold selectivity over PDE10A, respectively [15,21]. More recently, we gained a further improvement in terms of in vitro PDE2A binding via replacement of the 1-methoxy in **TA1** by 1-(2-fluoroethoxy), resulting in the compound **TA5**, (Figure 1) [15,21,22]. However, after successful ^18^F-labeling, the obtained tracers, [^18^F]**TA3** and [^18^F]**TA4**, did not entirely succeed and were proven to be suitable only for in vitro autoradiographic PDE2A imaging. Beyond that, [^18^F]**TA5** failed to demonstrate specific binding in vitro [22]. The formation of brain-penetrating radiometabolites due to the O-defluoroalkylation limited their application for in vivo PDE2A imaging. Therefore, further structural modifications of tricyclic lead are inevitably required to allow appropriate in vivo PDE2A imaging [21,22]. A further tracer, [^18^F]**AQ28A**, obtained as a developmental compound from our PDE10 program (**AQ28A**, Figure 1), was proven to be sufficiently metabolically stable and to enable in vivo imaging of PDE10A in rodents by PET [24]. As one feature, the fluorinated aromatic ring of this compound was ascribed to its performance as a promising tracer. 

**Figure 2:**
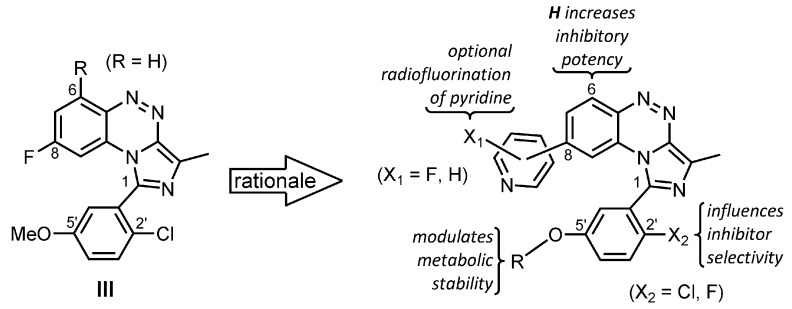
Approach for structural modification of the lead structure **III**.

As part of our ongoing commitment in ^18^F-PET tracer development devoted to PDE imaging (2A, 5A, and 10A) [21,22,24,29], we focused on Benzoimidazotriazine (BIT) as the scaffold, which differs in a benzo ring formally replacing the pyrido ring of our hitherto investigated **TA** compounds. Because of our findings from **AQ28A**, we felt encouraged to combine a 2-fluorpyridine moiety as a fluorine-bearing group with structural features required for PDE2A binding (Figure 2). Herein, we report the synthesis and in vitro evaluation of fluorinated derivatives containing a BIT scaffold and the selection of a promising ligand for the development of a ^18^F-labeled ligand for PDE2A imaging with PET. The use of a benzo fused imidazo[2,1-*c*][1,2,4]triazine allows us to readily perform structural modification in the six-membered benzo ring (8-position) while retaining the structural modifiability of the imidazole portion (1-position) of the tricycle (Figure 2). An example of this BIT series, compound **III** (Figure 1 and Figure 2), bearing no substituent at the 6-position, was more potent to PDE2A in comparison to **II** and served as the lead in our studies [26].

Therefore, our strategy was to modify this structure by changing the substituents at 1-(phenyl) and the 8-position (pyridinyl instead of F) in order to obtain potent and selective PDE2A inhibitors (Figure 2). These can be established via a standard palladium (Pd)-catalyzed Suzuki coupling reaction [30]. Our main interest was to obtain fluorinated compounds for the development of PDE2A PET tracers. Therefore, the introduction of fluorine-containing groups particularly at the 8-position (pyridinyl) is required. According to our previous work, several fluorinated pyridinyl substituents may enhance the potency and selectivity to PDE2A, and the incorporation of the pyridine moiety is tolerable. Besides, pyridine, as an electron-deficient aromatic ring, offers a convenient way to perform radiofluorination via nucleophilic aromatic substitution [31]. Furthermore, fluorinated pyridinyl substituents are relatively more stable towards metabolic degradation compared with fluoroalkyl groups, which were found to be more prone to defluoroalkylation [15,21,22,24].

## 2. Results and Discussion

### 2.1. Chemistry

**Scheme 1:**
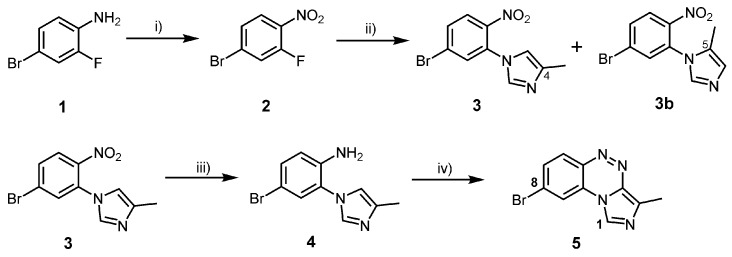
Synthesis of Compound **5**. (i) Oxidation, NaBO_3_·4H_2_O, acetic acid, 65 °C, 2–3 d; (ii) 4-methyl-1*H*-imidazole, K_2_CO_3_, DMF, room temperature (rt), 1–2 d; (iii) Fe (5 eq), HOAc/EtOH, reflux, 2.5 h; (iv) NaNO_2_, acetic acid, H_2_O, rt, 1 h.

The first of our synthetic approaches focused on bromo-3-methylbenzo[*e*]imidazo[5,1-*c*][1,2,4]triazine (Scheme 1, Compound **5**) as the key intermediate for further conversions into the desired final products (BIT derivatives). The 8-bromo-substituted tricycle **5** was synthesized in four steps starting from 4-bromo-2-fluoro-aniline (**1**) as depicted in Scheme 1.

The oxidation of the aromatic amino group with sodium perborate tetrahydrate (NaBO_3_·4H_2_O) in acetic acid afforded nitro compound **2** in a 51% yield. To avoid significant by-product formation, the reaction temperature was kept below 65 °C, and the aniline **1** was slowly added to the oxidizing agent [23]. The obtained 4-bromo-2-fluoro-nitrobenzene (**2**) was reacted with 4-methyl-1*H*-imidazole to provide the corresponding *N*-aryl-4- and *N*-aryl-5-methylimidazoles **3** and **3b**. This nucleophilic aromatic substitution (S_N_Ar) employed 1.2 eq of 4-Me-imidazole and 2.0 eq of K_2_CO_3_ in DMF and formed a mixture of **3** and **3b** in a ratio of ~4:1 [32,33]. However, the main regioisomer **3** could be purified via repeated recrystallization from methanol (50% yield). The reduction of **3** using iron afforded aniline **4** in high yield (92%). The iron powder represents a preferred reducing agent for nitro compounds bearing sensitive functional groups such as halides and/or other reducible groups [34]. A subsequent one-pot diazotation-intramolecular azo coupling of compound **4** by use of aqueous sodium nitrite in acetic acid gave the intermediate **5** in a satisfactory yield of 93%. The overall yield was 22%.

**Scheme 2:**
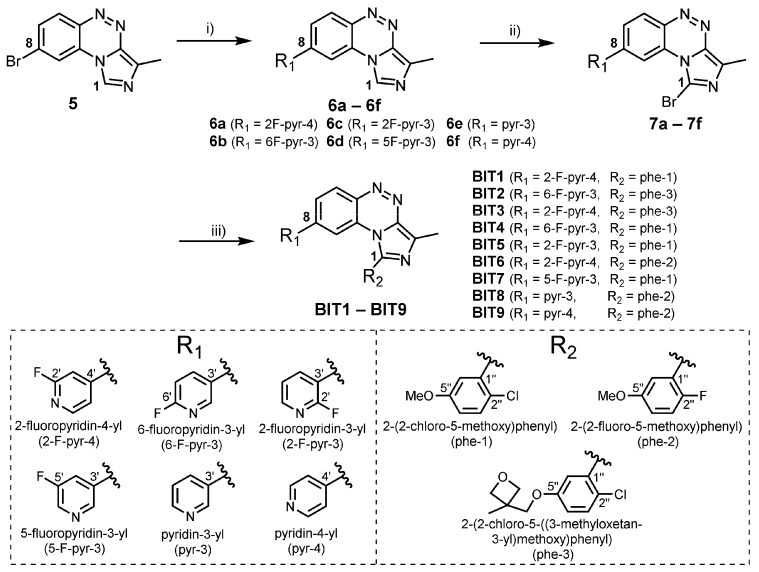
Approach to achieve a series of nine BIT derivatives. (i) R_1_B(OH)_2_ (**8a**–**8f**), K_2_CO_3_, Pd(PPh_3_)_4_, 1,4-dioxane:H_2_O (4:1), reflux, 1–2 d; (ii) NBS, MeCN, rt 4–6 h; (iii) R_2_B(OH)_2_ (**9a** [phe-1-B(OH)_2_], **9b** [phe-2-B(OH)_2_]), or R_2_B(pin) (**9c**, [phe-3-B(pin)]), K_2_CO_3_, Pd(PPh_3_)_4_, dioxane:H_2_O (4:1), reflux, 1–2 d.

The synthesis of diaryl-substituted compounds (BIT derivatives) starting from **5** is depicted in Scheme 2. Positions 1 and 8 were substituted with different aryl moieties by two independent Suzuki couplings, possible through an intermediate bromination on position 1. The first Suzuki coupling was used to introduce a substituted pyridine ring to the 8-position. Six different Boronic Acids (BAs), including fluoropyridinyl boronic acids (**8a**–**8d**) and pyridinyl boronic acids (**8e** and **8f**) as well, were coupled in the presence of K_2_CO_3_ as a base and Pd(Ph_3_)_4_ as a catalyst under standard conditions (Scheme 1, Table 1). 

Cross-coupling products **6a**–**6f** were obtained after flash column chromatography purification in yields from 28%–98%, as depicted in Table 1. Afterwards, the bromination of the imidazo fused ring in the 1-position was carried out using *N*-bromosuccinimide (NBS) in acetonitrile at room temperature and provided Compounds **7a**–**7f** in moderate to high yields (Table 1).

**Scheme 3:**
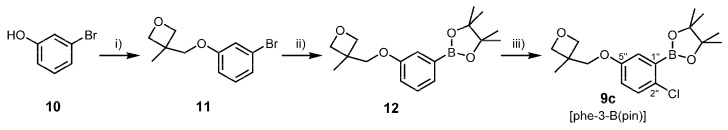
Preparation of **9c**. (i) 3-methyloxetan-3-yl-methyl-mesylate, Cs_2_CO_3_, K_2_CO_3_, MeCN, 75–79 °C, 18 h; (ii) B_2_pin_2_, PdCl_2_(dppf), KOAc, 2-Me-THF, Ar; (iii) NCS, DMF, rt.

By a second Suzuki coupling, the five-membered imidazole portion was functionalized (Scheme 2). Initially, two *ortho*-chlorophenyl boronic acid (BA) derivatives **9a** and **9c** were used for coupling with **7a** and **7b**, as well. In addition, to include the 5″-methoxy substituted BA **9a,** we investigated a derivative with a modified propoxy substitution by using **9c** with a (3-methyloxetan-3-yl)methoxy moiety at the C-5″-position (Scheme 2, **9c** [phe-3-B(pin)]). It has been reported that an oxetane residue may modulate and positively influence physicochemical, as well as biological properties of a drug candidate [35,36]. Besides, an oxetane moiety is regarded to induce stability towards metabolic attack similar to a corresponding geminal dimethyl unit (gem-Me_2_) [37], but in contrast will not increase the lipophilicity of the compound [35,36]. To make this oxetane derivative available, we prepared boronic ester **9c** in three steps, starting from 3-bromo-phenol (**10**) (Scheme 3). 

First, phenol **10** was reacted with 3-methyloxetan-3-yl-methyl-mesylate to provide aryl ether **11** (85% yield). Afterwards, Miyaura-borylation was performed to react **11** with bis(pinacolato)diboron (B_2_pin_2_) in the presence of PdCl_2_(dppf) and KOAc as a catalyst and base, respectively, providing boronate ester **12** in a good yield of 81%. Finally, the chlorination of **12** ortho to the boronate ester by means of *N*-chloro-succinimide (NCS) in DMF at room temperature afforded **9c** in a yield of 56% [38]. 

Boronic acid derivatives **9a** and **9c** were reacted with **7a** and **7b** via cross-coupling under conditions similar to those described for the reactions of BAs **8a**–**8f** with compound **5**. Reaction products **BIT1**, **BIT2**, and **BIT3** were obtained in satisfactory yields, as depicted in Table 2 (Entries 1–3). 

In vitro evaluation data of the first series of final compounds revealed **BIT1** to have a higher inhibitory potency towards PDE2A in contrast to **BIT2** and **BIT3** (see the In Vitro Evaluation section). Therefore, on the basis of this first finding, we prepared further BIT derivatives and investigated the effect of an alteration of the pyridinyl substitution in 8-position on the PDE2A inhibition. For that purpose, we synthesized **BIT4**–**BIT9** with the methoxy group in the 5′-position, since this substitution appeared to be the most promising for the inhibition of PDE2A. Of the newly-synthesized compounds, **BIT6**, **BIT8**, and **BIT9** possessed the fluoro substitution in position 2′ of the phenyl residue. **BIT4**–**BIT9** were obtained in moderate to good yields as shown in Table 2 (Entries 4–9). After characterization by one- and two-dimensional NMR spectroscopy and HR-MS (see Supplementary Materials), the new derivatives were evaluated in vitro (see the In Vitro Evaluation Section).

### 2.2. In Vitro Evaluation: Structure-Activity Relationships of BIT Derivatives 

Final products (BIT derivatives) were evaluated in vitro towards PDEs by means of radioligand binding assays. Inhibition of PDE2A was measured at an inhibitor concentration of **10 nM** and those of other PDEs at a concentration of **1 µM** for each BIT derivative. The inhibitory potencies of all BIT compounds are summarized in Table 3. 

We first focused our attention on modifications at the 1-position while keeping the 2-fluoropyridin-4-yl (2-F-pyr-4) fixed at the 8-position as for compounds **BIT1** and **BIT3**. According to our previous work [21], a longer alkyl side chain length (3-fluoropropoxy to 4-fluorobutoxy, as **TA3**–**TA4**) contributes to improved potency, as well as selectivity towards PDE2A, as was also shown for **TA1** (Figure 1) [15,21]. However, the substitution of the 5′-methoxy group (**BIT1**) by a (3-methyloxetan-3-yl)methoxy moiety (**BIT3**) in the 1-phenyl moiety resulted in a strong reduction of inhibitory potency towards PDE2A3 from 82.9% to 13.2% (at 10 nM). One possible explanation for that result might be the increased polarity of the chosen oxetanyl-methoxy residue compared with alkoxy groups, which possibly is unfavorable for key interactions towards PDE2A. It was reported that the phenylpropyl portion in the imidazole ring of **BAY 60-7550** interacts with Leu770, one of the amino acids that is known to be responsible for hydrophobic pocket (H-pocket) formation in the PDE2A active site [39]. Moreover, **BAY 60-7550** binding to the PDE2A active site suggested that the selectivity for PDE2A was generated from the ability of the ligand to induce a conformational change of Leu770 [1,39]. Therefore, it is hypothesized that **BIT3** may not induce the conformational change of the Leu770 amino acid that generates the selectivity pocket formation, thus leading to potency losses towards PDE2A [1,39].

In the case of a pair of 6-F-pyr-3-substituted products, **BIT2** and **BIT4**, a weak inverse effect of PDE2A binding was observed [40], depending on the nature of the 5″ substituent and in comparison to **BIT3**/**BIT1**. Strikingly, the much lower inhibition displayed by **BIT4** (2.63%) in contrast to **BIT1** (82.9%), bearing the same phe-1 substituent, points to the impact of the pyridine substitution pattern in exerting inhibition on PDE2A.

In this connection, another factor that contributes to the selectivity of **BAY 60-7550** is a glutamine-switch mechanism [39], which is generally proposed for dual substrate-specific PDEs, such as PDE2A [1,39]. We assume that triazine N-5 in our scaffold might accept a hydrogen bond from Gln859 in a similar mode as described for the pyrazine portion in bioisosteric triazoloquinoxaline-based PDE2A inhibitors [16,20]. We further investigated the influence of different pyridinyl substituents at the 8-position on PDE2A inhibition while keeping the phe-1 fixed at the 1-position. In addition to **BIT4,** containing a 6-F-pyr-3, the PDE2A inhibition also dropped significantly with **BIT5**, containing a 2-F-pyr-3 at the 8-position. Interestingly, a potency of at least 50% remained with the introduction of the 5-F-pyr-3 residue (**BIT7**) at the 8-position. It is not unambiguously clear whether this was caused only by the position of the pyridine nitrogen, which may affect rotational freedom of the ring *via* active site H-bonding to the pyridine N [16], or in the case of **BIT1** and **BIT7**, additionally by X‒H∙∙∙F‒C interactions with fluorine [41,42]. Considering this, we found an influence of substituent variation at the 8-position while keeping phe-1 at the 1-position in decreasing order of activity towards PDE2A as 2-F-pyr-4 > 5-F-pyr-3 > 6-F-pyr-3 > 2-F-pyr-3. It was supposed that 2-F-pyr-4 and 5-F-pyr-3 likely maintained the conformational locking by the H-bond, resulting in higher PDE2A potency of **BIT1** and **BIT7**. Moreover, compared to **BIT7**, both **BIT4** and **BIT5** demonstrated eight- and four-fold selectivity over PDE10A, respectively. 

We then directed our attention to investigate the influence of ortho-fluorophenyl (phe-2) at the 1-position. In the case of **BIT6**, having a 2-F-pyr-4 at the 8-position, a weakly lower potency towards PDE2A of 56% was observed, when compared to **BIT1**, which is in accordance with the known positive PDE2A inhibitory effect of 2″-Cl in comparison to 2″-F [20,28]. A non-fluorinated pyridine (pyr-4 in **BIT9**) also maintained the inhibitory activity towards PDE2A of 80.5%, close to **BIT1** (82.9%), however at the expense of increasing inhibition towards PDE10A and PDE4A (96.6% and 86.8% at a 1 μM inhibitor concentration) in contrast to **BIT1** (32.4% and 58.6% at 1 μM). Incorporation of pyr-3 resulted in a significant loss of PDE2A inhibition, which is consistent with the result of **BIT2**, **BIT4**, and **BIT5,** having also a substituted pyridin-3-yl at the 8-position, but not with **BIT7**, which may be due to reasons discussed above. Again, the strong effect of the N-atom position in the pyridine ring on PDE2A inhibition may be explained by differing conformational preferences between pyr-4 and pyr-3 [11]. It was assumed that pyr-3 allows more rotational freedom, resulting in energy loss of binding [16]. The selectivity towards certain PDEs was decreased when exchanging phe-1 with phe-2. Thus, in addition to lower potency towards PDE2A, **BIT6** displayed higher inhibition of PDE10A when compared to **BIT1** (74% vs. 32%, at 1 µM). Therefore, these results suggest that the ortho-chlorophenyl (phe-1) at 1-position was useful for PDE2A potency and selectivity. 

Three compounds, **BIT1**, **BIT6**, and **BIT9**, were selected for estimation of IC_50_ values of PDE2A and PDE10A inhibition, to determine the potency in more detail and also the PDE10A/PDE2A selectivity ratio. The related IC_50_ values are shown in Table 4.

Finally, the potencies of **BIT1** towards a range of other PDEs were evaluated as depicted in Table 5. **BIT1** showed a good selectivity over other PDEs (12.6%–23.3% for other PDEs, measured at 1 µM). Summarizing, **BIT1** had a good selectivity over PDE10A (16-fold), while **BIT6** and **BIT9** had 2.5-fold and 1.1-fold selectivity over PDE10A, respectively. Applying similar assay conditions, **TA1** and **BAY 60-7550** displayed an IC_50_ value towards PDE2A of 10.8 nM and 0.66 nM, respectively. Thus, **BIT1,** possessing phe-1 and 2-F-pyr-4 at the 1- and 8-positions, proved to be a prospective ligand for labeling with ^18^F for PDE2A PET imaging. 

### 2.3. Incubations with Mouse Liver Microsomes 

To gain first information about whether **BIT1** is prone to metabolic degradation, the compound was investigated in an in vitro test system. Briefly, **BIT1** was incubated with Mouse Liver Microsomes (MLM), in the presence of NADPH, at 37 °C for 90 min [43]. After protein precipitation by addition of acetonitrile and subsequent centrifugation, the supernatant was examined by HPLC-UV-MS. 

**Figure 3:**
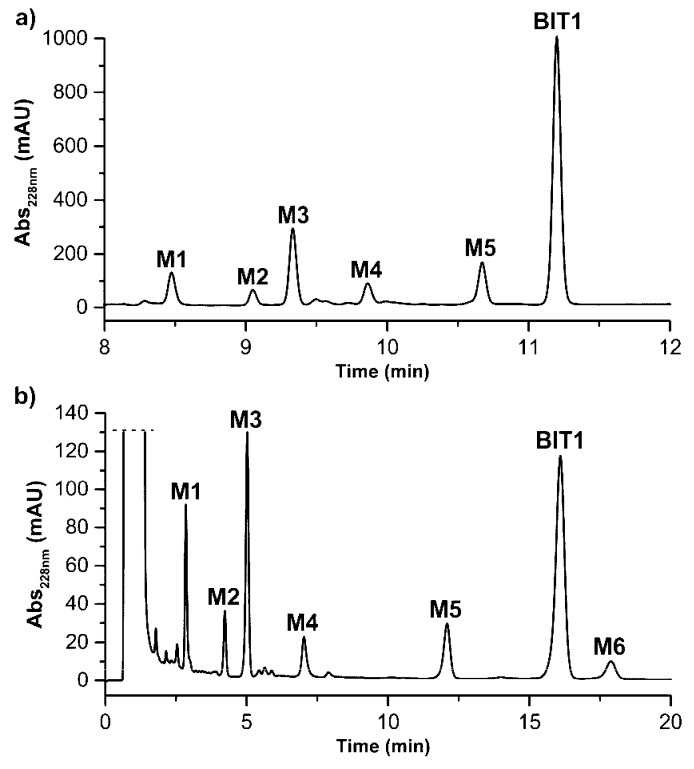
HPLC-UV chromatogram after incubation of **BIT1** with MLM in the presence of NADPH (for conditions, see the experimental part): (**a**) UV chromatogram (partial) recorded during gradient elution (Method A); (**b**) UV chromatogram recorded during isocratic elution (Method B).

After incubation with MLM for 90 min, unchanged **BIT1** could still be detected in high amounts (Figure 3). The main fractions of in vitro metabolites were products of a mono-oxygenation of **BIT1**, namely Metabolites **M3**, **M5**, and **M6** (see Table 6). 

The metabolite **M6** was detectable only by an HPLC method with isocratic elution as shown in Figure 3b. Furthermore, one metabolite resulting from a two-fold oxygenation (**M2**) was found. Due to the limitations of a single quadrupole MS, it was not possible to obtain information regarding the sites of functionalization in the molecule, neither to distinguish products from *N*-oxidation or *C*-hydroxylation [44]. In contrast, for the formation of **M1** and **M4** by reduction and demethylation, respectively, the chloro(methoxy)-phenyl moiety is expected to undergo these reactions under the conditions applied.

The considerable metabolic stability of **BIT1** in vitro supports the potential suitability of the corresponding ^18^F-labelled compounds as a radioligand for PET. However, due to the retention properties of the metabolite **M6** in HPLC this metabolite may have a similar or higher lipophilicity in comparison to **BIT1**. Therefore, of the metabolites detected, **M6** most likely bears the risk of passing the blood-brain barrier and influencing brain PET results. Hence, future in vivo metabolism studies should pay attention to the occurrence of this metabolite, as well as to distinguish it chromatographically from the parent compound.

## 3. Materials and Methods 

### 3.1. General Information

Chemicals were purchased from following suppliers: Manchester Organics, abcr, VWR, Fluka, Acros, Roth, ChemPure, Merck, Sigma Aldrich, Apollo scientific, and Fluorochem. Solvents were dried before use, if required. Air- and moisture-sensitive reactions were carried out under argon atmosphere. Room temperature (rt) refers to 20–25 °C. The progress of a reaction was monitored by thin layer chromatography using pre-coated TLC sheets POLYGRAM^®^ SIL G/UV254 purchased from Macherey-Nagel. Detected spots were observed under UV light at λ 254 nm and 365 nm. Flash chromatography was performed with silica gel 40–63 μm from VWR Chemicals.

NMR spectra (^1^H, ^13^C, ^19^F) were recorded on Mercury 300/Mercury 400 (Varian, Palo Alto, CA, USA) or Fourier 300/Avance DRX 400 Bruker (Billerica, MA, USA) instruments. Signals of solvents were used as internal standards for ^1^H-NMR (CDCl_3_, δ_H_ = 7.26; DMSO-*d*_6_, δ_H_ = 2.50) and ^13^C-NMR (CDCl_3_, δ_C_ = 77.16; DMSO-*d*_6_, δ_C_ = 39.52). The chemical shifts (δ) are reported in ppm as follows: s, singlet; d, doublet; t, triplet; m, multiplet; and the coupling constants (J) are reported in Hz. Mass spectra were recorded on an ESQUIRE 3000 Plus (ESI, low resolution) and a 7 T APEX II (ESI, high resolution) from Bruker Daltonics. 

### 3.2. Syntheses

*4-Bromo-2-fluoro-nitrobenzene* (**2**): A suspension of sodium perborate tetrahydrate (20.24 g, 0.13 mol) in glacial acetic acid (80 mL) was stirred at 65 °C. 4-Bromo-2-fluoro-aniline (5 g, 26.31 mmol, 1 eq) in 35 mL acetic acid was dropwise added over 5 h. The reaction was heated overnight, and subsequently, another portion of NaBO_3_·4H_2_O (12.2 g, 78.9 mmol) was added. After full consumption of the starting material, the reaction mixture was cooled to room temperature, the solid filtered off, and the filtrate quenched with ice-cold water (600 mL). Then, the precipitate was filtrated and purified with column chromatography (hexane/chloroform, 2:1) to give the product as yellow solid 2 (2.96 g, 51%). TLC (hexane/CHCl_3_ (2:1)): R_f_ = 0.32. ^1^H-NMR (400 MHz, CDCl_3_) δ_H_ = 8.01–7.93 (m, 1H), 7.50 (dd, *J* = 10.1, 1.9 Hz, 1H), 7.48–7.44 (m, 1H). ^13^C-NMR (101 MHz, CDCl_3_) δ_C_ = 155.48 (d, *J* = 269.9 Hz), 136.60 (d, *J* = 7.2 Hz), 129.56 (d, *J* = 8.9 Hz), 128.24 (d, *J* = 4.3 Hz), 127.28 (d, *J* = 2.5 Hz), 122.21 (d, *J* = 23.6 Hz). LR-MS (EI): *m/z* = 219 (calcd. 219 for C_6_H_3_^79^BrFNO_2_^+^ [M]^+^)

*4-Bromo-2-(4-methyl-1H-imidazol-1-yl) nitrobenzene* (**3**): A mixture of compound **2** (9.46 g, 43 mmol) and K_2_CO_3_ (11.9 g, 86 mmol) in DMF (40 mL) was stirred at 4 °C, while a solution of 4-methyl imidazole (3.78 g, 46 mmol) in DMF (10 mL) was slowly added in the course of 2 h. Afterwards, stirring was continued for 10 h at room temperature. The reaction mixture was poured into water (250 mL), and the formed precipitate was filtered off, washed, and dried to give a brown yellow solid (10.47 g), which was found to be an impure mixture of regioisomeric Products **3** and **3b** in a ratio of ~4:1, according to ^1^H-NMR. The solid was dissolved in CHCl_3_ (80 mL) and filtered through a plug of silica gel (2 g). The solvent was evaporated, and the remaining solid was recrystallized twice from aqueous EtOH (80%) to give pure 3, as bright yellow crystals (7.09 g, 58%). Mp. 151–152.5 °C; TLC (CHCl_3_/MeCN (10:1)]: R_f_ = 0.33. ^1^H-NMR (400 MHz, CDCl_3_) δ_H_ = 7.85 (d, *J* = 8.7 Hz, 1H), 7.70 (dd, *J* = 8.7, 2.0 Hz, 1H), 7.61 (d, *J* = 2.0 Hz, 1H), 7.52 (d, *J* = 0.9 Hz, 1H), 6.75 (s, 1H), 2.27 (d, *J* = 0.9 Hz, 3H). ^13^C-NMR (101 MHz, CDCl_3_) δ_C_ = 143.91, 140.24, 136.31, 132.37, 131.95, 131.47, 127.95, 126.68, 116.35, 13.71. LR-MS (ESI+): *m/z* = 282, (calcd. 282 for C_10_H_9_^79^BrN_3_O_2_^+^ [M + H]^+^).

The minor regioisomer was isolated from a sample (0.36 g) of the filtrate obtained from the first recrystallization. It was purified via flash purification applying a gradient from CHCl_3_ (100%) to CHCl_3_/MeCN (30:1) to give pure **3b**, as a yellow solid (0.11 g). Mp. 107.5–109 °C; TLC [CHCl_3_/MeCN (10:1)]: R_f_ = 0.26. ^1^H-NMR (400 MHz, CDCl_3_) δ_H_ = 7.97 (d, *J* = 8.7 Hz, 1H), 7.81 (dd, *J* = 8.7, 2.1 Hz, 1H), 7.59 (d, *J* = 2.1 Hz, 1H), 7.46 (s, 1H), 6.91 (s, 1H), 2.04 (s, 3H). ^13^C-NMR (101 MHz, CDCl_3_) δ_C_ = 145.38, 136.78, 133.60, 133.54, 131.16, 128.99, 128.22, 127.82, 126.78, 9.24.

*4-Bromo-2-(4-methyl-1 H-imidazol-1-yl) aniline* (**4**): Nitro compound **3** (4.42 g, 15.7 mmol, 1 eq) was dissolved under argon in a 1:1 mixture of ethanol (50 mL) and acetic acid (50 mL). Iron powder (5.25 g, 94 mmol) was added, and the reaction mixture was stirred under reflux for 2.5 h (bath temperature 115–120 °C). The mixture was filtered through celite, and the filtrate was adjusted to pH 8 with Na_2_CO_3_ until a greenish solid formed. The mixture was extracted with Ethyl Acetate (EE, 3 × 100 mL), and the organic phase was washed with water (2 × 100 mL) and saturated NaCl solution (100 mL), and dried (Na_2_SO_4_)_._ The solvent was evaporated to give a beige solid (3.64 g, 92%). TLC (CHCl_3_/MeOH/30% NH_3_ (10:1:0.1)): R_f_ = 0.41. ^1^H-NMR (400 MHz, CDCl_3_) δ_H_ = 7.52 (d, *J* = 1.0 Hz, 1H), 7.30 (dd, *J* = 8.6, 2.3 Hz, 1H), 7.23 (d, *J* = 2.2 Hz, 1H), 6.80 (s, 1H), 6.71 (t, *J* = 8.0 Hz, 1H), 3.68 (d, *J* = 48.0 Hz, 2H), 2.30 (d, *J* = 0.6 Hz, 3H).^13^C-NMR (101 MHz, CDCl_3_) δ_C_ = 141.22, 139.48, 136.72, 132.47, 129.50, 124.52, 117.94, 116.32, 109.38, 13.77. LR-MS (ESI+): *m/z* = 274, (calcd. 274 for C_10_H_10_^79^BrN_3_Na^+^ [M + Na]^+^).

*8-Bromo-3-methylbenzo[e]imidazo[5,1-c][1,2,4]triazine* (**5**): A solution of NaNO_2_ (1.28 g, 18.6 mmol) in H_2_O (10 mL) was added to a stirred solution of 4 (3.13 g, 12.4 mmol) in acetic acid (60 mL) at room temperature. A yellow precipitate formed immediately. After 5 h of stirring, the reaction mixture was evaporated to half of its original volume and diluted with H_2_O (50 mL). The pH was carefully adjusted to pH 8 by the addition of solid NaHCO_3_. The yellow solid was filtered off and dried to give **5** (3.24 g, 98% sufficiently pure for the next step). The solid was dissolved in CHCl_3_ (150 mL) and filtered through a plug of silica gel (2 g). The filtrate was concentrated and triturated with CHCl_3_/heptane mixtures to afford a yellow powder (2.9 g, 89%). TLC (hexane/ethyl acetate (1:3)): R_f_ = 0.45. ^1^H-NMR (400 MHz, DMSO-*d*_6_) δ_H_ = 9.16 (s, 1H), 8.70 (d, *J* = 2.0 Hz, 1H), 8.29 (d, *J* = 8.6 Hz, 1H), 7.88 (dd, *J* = 8.6, 2.0 Hz, 1H), 2.74 (s, 3H). ^13^C-NMR (101 MHz, DMSO-*d*_6_) δ_C_ = 137.71, 135.87, 134.37, 131.40, 130.55, 127.23, 126.01, 123.04, 118.35, 12.39. LR-MS (ESI+): *m/z* = 263 (calcd. 263 for C_10_H_8_^79^BrN_4_^+^ [M + H]^+^).

#### 3.2.1. General Procedure A for the Suzuki Coupling of Bromo Derivatives **5**, **7a**, **7b**, **7c**, **7d**, **7e**, and **7f**

Brominated compound (1 eq), boronic acid derivative (BA, **8a**–**8f**, **9a**–**9c**, 1.1–1.3 eq), and K_2_CO_3_ (2–3 eq) were combined in a mixture of 1,4-dioxane and water (4:1). The suspension was degassed with argon for 5–10 min, and Pd(PPh_3_)_4_ (5–10 mol%) was added. The mixture was refluxed (bath temperature 102–108 °C) for 1–2 days until completion of the reaction (as indicated by TLC). The solvent was removed, and the residue was partitioned between CHCl_3_ and water. The aqueous layer was extracted twice with CHCl_3_, and the combined organic layers were dried (Na_2_SO_4_) and the solvent evaporated. Unless stated otherwise, the obtained residue was purified by flash chromatography on silica gel using CHCl_3_/MeOH (10:1) as the eluent. The following products were isolated:

*8-(2-Fluoropyridin-4-yl)-3-methylbenzo[e]imidazo[5,1-c][1,2,4]triazine* (**6a**): Based on General Procedure A of Suzuki coupling, a mixture of compound **5** (101 mg, 0.38 mmol,1 eq), BA **8a** (65 mg,0.46 mmol, 1.2 eq), and K_2_CO_3_ (131 mg, 0.95 mmol, 2.5 eq), in dioxane/water (5 mL) were reacted in the presence of Pd(PPh_3_)_4_ (44 mg, 10 mol%) to give after purification compound **6a** (81 mg, 76%) as a yellow solid. TLC (hexane/ethyl acetate (1:1)): R_f_ = 0.25. ^1^H-NMR (400 MHz, DMSO-*d*_6_) δ_H_ = 9.28 (s, 1H), 8.92 (d, *J* = 1.9 Hz, 1H), 8.52 (d, *J* = 8.5 Hz, 1H), 8.46 (d, *J* = 5.3 Hz, 1H), 8.25 (dd, *J* = 8.5, 1.9 Hz, 1H), 7.97 (dt, *J* = 5.3, 1.8 Hz, 1H), 7.82 (s, 1H), 2.79 (s, 3H). LR-MS (ESI+): *m/z* = 280, (calcd. 280 for C_15_H_11_FN_5_^+^ [M + H]^+^).

*8-(6-Fluoropyridin-3-yl)-3-methylbenzo[e]imidazo[5,1-c][1,2,4]triazine* (**6b**): According to General Procedure A, compound **5** (500 mg, 1.9 mmol,1 eq), BA **8b** (321 mg, 2.28 mmol, 1.2 eq), K_2_CO_3_ (788 mg, 5.7 mmol, 3 eq), and Pd(PPh_3_)_4_ (220 mg, 10 mol%) were reacted in dioxane/water (24 mL) to yield after purification compound **6b** (150 mg, 28%), as a yellow powder. TLC (CH_3_Cl_3_/MeOH (10:1)): R_f_ = 0.38. ^1^H-NMR (400 MHz, CDCl_3_) δ_H_ = 8.62–8.52 (m, 3H), 8.12 (ddd, *J* = 8.4, 7.4, 2.7 Hz, 1H), 7.98 (d, *J* = 1.8 Hz, 1H), 7.82 (dd, *J* = 8.4, 1.8 Hz, 1H), 7.13 (dd, *J* = 8.5, 3.1 Hz, 1H), 2.91 (s, 3H). LR-MS (ESI+): *m/z* = 280, (calcd. 280 for C_15_H_11_FN_5_^+^ [M + H]^+^).

*8-(2-Fluoropyridin-3-yl)-3-methylbenzo[e]imidazo[5,1-c][1,2,4]triazine* (**6c**): According to General Procedure A, compound **5** (501 mg, 1.9 mmol, 1 eq), BA **8c** (325 mg, 2.28 mmol, 1.2 eq), K_2_CO_3_ (661 mg, 4.75 mmol, 2.5 eq), and Pd(PPh_3_)_4_ (219 mg, 10 mol%) were reacted in dioxane/water (15 mL) to afford after purification compound 6c (0.52 g, 98%), as a yellow solid. TLC (CHCl_3_/MeOH (10:1)): R_f_ = 0.38. ^1^H-NMR (400 MHz, CDCl_3_) δ_H_ = 8.58–8.52 (m, 1H), 8.34 (dt, *J* = 5.0, 1.5 Hz, 1H), 8.10–7.99 (m, 1H), 7.85 (dd, *J* = 8.4, 1.6 Hz, 1H), 7.71–7.62 (m, 1H), 7.58–7.37 (m, 2H), 2.92 (s, 3H). LR-MS (ESI+): *m/z* = 280 (calcd. 280 for C_15_H_11_FN_5_^+^ [M + H]^+^).

*8-(5-Fuoropyridin-3-yl)-3-methylbenzo[e]imidazo[5,1-c][1,2,4]triazine* (**6d**): According to General Procedure A, compound **5** (200 mg, 0.76 mmol, 1 eq), BA **8d** (118 mg, 0.84 mmol, 1.1 eq), K_2_CO_3_ (315 mg, 2.28 mmol, 3.0 eq), and Pd(PPh_3_)_4_ (44 mg, 5 mol%) were reacted in dioxane/water (10 mL) to give after purification Compound **6d** (0.12 g, 56%), as a yellow powder. TLC (CHCl_3_/MeOH (10:1)): R_f_ = 0.29. ^1^H-NMR (400 MHz, DMSO-*d*_6_) δ_H_ = 9.26 (s, 1H), 9.09 (d, *J* = 1.9 Hz, 1H), 8.88 (d, *J* = 1.8 Hz, 1H), 8.71 (d, *J* = 2.7 Hz, 1H), 8.49 (d, *J* = 8.5 Hz, 1H), 8.36 (dt, *J* = 10.3, 2.3 Hz, 1H), 8.19 (dd, *J* = 8.5, 1.9 Hz, 1H), 2.77 (s, 3H).

*3-Methyl-8-(pyridin-3-yl)benzo[e]imidazo[5,1-c][1,2,4]triazine* (**6e**): According to General Procedure A, compound **5** (1.0 g, 3.8 mmol, 1 eq), BA **8e** (0.61 mg, 4.94 mmol, 1.3 eq), K_2_CO_3_ (1.58 g, 11.4 mmol, 3 eq), and Pd(PPh_3_)_4_ (220 mg, 5 mol%) were reacted in dioxane/water (50 mL) to give after purification by flash chromatography compound **6e** (0.76 g, 77%), as a yellow solid. TLC (CHCl_3_/MeOH (10:1)): R_f_ = 0.20. ^1^H-NMR (400 MHz, DMSO-*d*_6_) δ_H_ = 9.23 (s, 1H), 9.16 (d, *J* = 2.1 Hz, 1H), 8.77 (d, *J* = 1.8 Hz, 1H), 8.69 (dd, *J* = 4.8, 1.6 Hz, 1H), 8.45 (d, *J* = 8.4 Hz, 1H), 8.35–8.30 (m, 1H), 8.11 (dd, *J* = 8.5, 1.8 Hz, 1H), 7.60 (ddd, *J* = 8.0, 4.8, 0.9 Hz, 1H), 2.75 (s, 3H). LR-MS (ESI+): *m/z* = 262, (calcd. 262 for C_15_H_12_N_5_^+^ [M+H]^+^).

*3-Methyl-8-(pyridin-4yl)benzo[e]imidazo[5,1-c][1,2,4]triazine* (**6f**): According to General Procedure A, compound **5** (200 mg, 0.76 mmol, 1 eq), K_2_CO_3_ (265 mg, 1.92 mmol, 2.52 eq), BA **8f** (119 mg, 0.968 mmol, 1.27 eq), and Pd(PPh_3_)_4_ (44 mg, 0.038 mmol, 5 mol%) were reacted in dioxane/water (5 mL) to afford after purification compound **6f** (173 mg, 87%), as a yellow solid. TLC (CHCl_3_/MeOH (10:1)): R_f_ = 0.23. ^1^H-NMR (300 MHz, DMSO-*d*_6_) δ_H_ = 9.26 (s, 1H), 8.82 (d, *J* = 1.8 Hz, 1H), 8.78–8.75 (m, 2H), 8.47 (d, *J* = 8.5 Hz, 1H), 8.15 (dd, *J* = 8.5, 1.9 Hz, 1H), 7.96–7.92 (m, 2H), 2.75 (s, 3H).

#### 3.2.2. General Procedure B for Bromination of Compounds **6a**, **6b**, **6c**, **6d**, **6e**, and **6f**

N-Bromosuccinimide (NBS, ~1.5 eq) was added to a suspension of compounds **6a**–**6f** in acetonitrile. The reaction mixture was protected from light and stirred at room temperature for 1–2 d. After the full conversion of the starting material, the mixture was partitioned between CHCl_3_ and H_2_O. The organic layer was washed with aqueous NaHCO_3_ (5%), water, and saturated NaCl solution. After drying (Na_2_SO_4_) and evaporation, the residue was purified by flash chromatography, eluting with CHCl_3_/MeOH (10:1).

*1-Bromo-8-(2-fluoropyridin-4-yl)methylbenzo[e]imidazo[5,1-c][1,2,4]triazine* (**7a**): According to General Procedure B, for bromination, compound **6a** (300 mg, 1.07 mmol) and NBS (287 mg, 1.61 mmol) were reacted in acetonitrile (20 mL) to give after purification compound **7a**, as a yellow solid (306 mg, 79%). TLC (CHCl_3_/MeOH (10:1)): R_f_ = 0.74. ^1^H-NMR (400 MHz, CDCl_3_) δ_H_ = 9.25 (t, *J* = 10.2 Hz, 1H), 8.59 (d, *J* = 8.4 Hz, 1H), 8.42 (t, *J* = 8.7 Hz, 1H), 7.94 (dd, *J* = 8.4, 1.8 Hz, 1H), 7.55–7.48 (m, 1H), 7.25 (s, 1H), 2.89 (d, *J* = 3.6 Hz, 3H). LR-MS (ESI+): *m/z* = 358 (calcd. 358 for C_15_H_10_^79^BrFN_5_^+^ [M + H]^+^) 

*1-Bromo-8-(6-fluoropyridin-3-yl)-3-methylbenzo[e]imidazo[5,1-c][1,2,4]triazine* (**7b**): According to General Procedure B, compound **6b** (354 mg, 1.27 mmol) and NBS (338 mg, 1.9 mmol) were reacted in acetonitrile (16 mL) to give after purification Compound 7b, as a yellow solid (290 mg, 63%). TLC (CHCl_3_/MeOH (10:1)): R_f_ = 0.71. ^1^H-NMR (400 MHz, CDCl_3_) δ_H_ = 9.15 (d, *J* = 1.8 Hz, 1H), 8.59 (d, *J* = 2.6 Hz, 1H), 8.56 (d, *J* = 8.4 Hz, 1H), 8.12 (ddd, *J* = 8.5, 7.4, 2.7 Hz, 1H), 7.87 (dd, *J* = 8.4, 1.8 Hz, 1H), 7.13 (dd, *J* = 8.5, 3.0 Hz, 1H), 2.88 (s, 3H). LR-MS (ESI+): *m/z* = 358 (calcd. 358 for C_15_H_10_^79^BrFN_5_^+^, [M + H]^+^).

*1-Bromo-8-(2-fluoropyridin-3-yl)-3-methylbenzo[e]imidazo[5,1-c][1,2,4]triazine* (**7c**): According To General Procedure B, compound **6c** (169 mg, 0.61 mmol) and NBS (162 mg, 0.91 mmol) were reacted in acetonitrile (6 mL) to give after purification compound **7c**, as a yellow solid (192 mg, 88%). TLC (CHCl_3_/MeOH (10:1)), R_f_ = 0.66. ^1^H-NMR (400 MHz, CDCl_3_) δ**_H_** = 9.23 (t, *J* = 1.5 Hz, 1H), 8.54 (d, *J* = 8.4 Hz, 1H), 8.34 (dt, *J* = 4.9, 1.5 Hz, 1H), 8.08–8.00 (m, 1H), 7.89 (dt, *J* = 8.4, 1.6 Hz, 1H), 7.46–7.37 (m, 1H), 2.88 (s, 3H). LR-MS (ESI+): *m/z* = 358, (calcd. 358 for C_15_H_10_^79^BrFN_5_^+^ [M + H]^+^).

*1-Bromo-8-(5-fluoropyridin-3-yl)-3-methylbenzo[e]imidazo[5,1-c][1,2,4]triazine* (**7d**): According to General Procedure B, compound **6d** (120 mg, 0.43 mmol) and NBS (115 mg, 0.65 mmol) were reacted in acetonitrile (4 mL) to afford after purification Compound **7d**, (151 mg, 98%), as a yellow solid. TLC (CHCl_3_/MeOH (10:1)), R_f_ = 0.71. ^1^H-NMR (300 MHz, CDCl_3_) δ_H_ = 9.20–9.19 (m, 1H), 8.83–8.81 (m, 1H), 8.61–8.59 (m, 1H), 8.56 (d, *J* = 0.4 Hz, 1H), 7.90 (dd, *J* = 8.4, 1.8 Hz, 1H), 7.73 (ddd, *J* = 9.1, 2.7, 1.9 Hz, 1H), 2.88 (s, 3H). LR-MS (ESI+): *m/z* = 358, (calcd. 358 for C_15_H_10_^79^BrFN_5_^+^ [M + H]^+^).

*1-Bromo-3-methyl-8-(pyridin-3-yl)benzo[e]imidazo[5,1-c][1,2,4]triazine* (**7e**): According to General Procedure B, compound **6e** (300 mg, 1.15 mmol, 1 eq) and NBS (306 mg, 1.72 mmol) were reacted in acetonitrile (8 mL) to afford after purification compound **7e** (343 mg, 87%), as a yellow solid. TLC (CHCl_3_/MeOH (10:1)): R_f_ = 0.47. ^1^H-NMR (400 MHz, CDCl_3_) δ_H_ = 9.19 (d, *J* = 1.7 Hz, 1H), 9.00 (dd, *J* = 2.5, 0.9 Hz, 1H), 8.73 (dd, *J* = 4.8, 1.6 Hz, 1H), 8.55 (d, *J* = 8.4 Hz, 1H), 8.03 (ddd, *J* = 8.0, 2.5, 1.6 Hz, 1H), 7.91 (dd, *J* = 8.4, 1.8 Hz, 1H), 7.50 (ddd, *J* = 8.0, 4.8, 0.9 Hz, 1H), 2.87 (s, 3H). LR-MS (ESI+): *m/z* = 340, (calcd. 340 for C_15_H_11_^79^BrN_5_^+^ [M + H]^+^).

*1-Bromo-3-methyl-8-(pyridin-4-yl)benzo[e]imidazo[5,1-c][1,2,4]triazine* (**7f**): According to General Procedure B, compound **6f** (100 mg, 0.383 mmol, 1 eq) and NBS (102 mg, 0.573 mmol) were reacted in MeCN (4 mL) to give after purification compound **7f** (120 mg, 92%), as a yellow powder. TLC (CHCl_3_/MeOH (10:1)): R_f_ = 0.54. ^1^H-NMR (400 MHz, DMSO-*d*_6_) δ_H_ = 9.27 (d, *J* = 1.8 Hz, 1H), 8.85–8.78 (m, 2H), 8.58 (d, *J* = 8.4 Hz, 1H), 8.24 (dd, *J* = 8.4, 1.8 Hz, 1H), 7.97–7.91 (m, 1H), 2.76 (s, 3H). LR-MS (ESI+): *m/z* = 340, (calcd. 340 for C_15_H_11_^79^BrN_5_^+^ [M + H]^+^).

#### 3.2.3. Synthesis of Oxetanyl Building Block **9c**

*3-((3-Bromophenoxy)methyl)-3-methyloxetane* (**11**): A mixture of (3-methyloxetan-3-yl) methanol (2.09 g, 20.4 mmol) and triethylamine (TEA, 3.2 mL, 23 mmol) in MeCN (8 mL) was stirred and cooled at 15–25 °C, while a solution of methanesulfonyl chloride (1.55 mL, 20 mmol) in MeCN (3 mL) was dropwise added in the course of 20 min. The mixture was stirred at 22 °C for 4 h and at 0–2 °C for 30 min. The precipitated TEA hydrochloride was filtered off and washed with MeCN (2 × 2.5 mL). To the filtrate was successively added 3-bromophenol (**10**, 3.46 g, 20 mmol) and Cs_2_CO_3_ (3.26 g, 10 mmol) along with K_2_CO_3_ (2.07 g, 15 mmol). The resulting suspension was stirred and heated at 75–79 °C for 18 h (progress monitored by TLC). Upon completion, the suspension was stirred with methyl *tert*-butyl ether (MTBE, 50 mL) for 2 h. The solid was filtered off and the filtrate washed with sodium hydroxide solution (0.8 M, 1 × 20, 2 × 10 mL) and dried (Na_2_CO_3_). The solvent was evaporated and the remaining oil bulb-to-bulb distilled (4 mbar, air bath 150–190 °C) to afford compound **11**, as a colorless oil (4.35 g, 85%). TLC (heptane/MTBE (3:2)): R_f_ = 0.4. ^1^H-NMR (400 MHz, CDCl_3_) δ_H_ = 7.18–7.12 (m, 1H), 7.12-7.08 (m, 2H), 6.87 (ddd, *J* = 8.1, 2.5, 1.3 Hz, 1H), 4.61 (d, *J* = 6.0 Hz, 2H), 4.46 (d, *J* = 6.0 Hz, 2H), 4.00 (s, 2H), 1.43 (s, 3H). ^13^C-NMR (101 MHz, CDCl_3_) δ_C_ = 159.86, 130.70, 124.24, 122.94, 117.97, 113.62, 79.81 (2C), 73.06, 39.74, 21.33.

*4,4,5,5-Tetramethyl-2-(3-((3-methyloxetan-3-yl) methoxy) phenyl)-1,3,2-dioxaborolane* (**12**): A mixture of 3-bromophenylether **11** (1.75 g, 6.8 mmol), KOAc (1.5 g, 15.3 mmol), and bis(pinacolato)diboron (1.77 g, 15 mmol, in 2-methyltetrahydrofuran (24 mL)) was degassed with argon for 10 min. After addition of Pd(dppf)Cl_2_ (0.07 g, 0.096 mmol), the mixture was refluxed for 7 h. Upon completion (monitored by TLC), CH_2_Cl_2_ (25 mL) was added, the resulting suspension stirred for 1 h, and the solid filtered off. The filtrate was evaporated, and the viscous residue (3.2 g) was dissolved in MTBE (70 mL) and subsequently filtered through a short silica gel column (H 2 cm × D 2 cm). The filtrate was extracted with aqueous NaOH (0.75 M, 4 × 12 mL). The alkaline extract was neutralized by slow addition of aqueous HCl (4 M) at 0–2 °C (→ pH 6–7). The separated oil was extracted with MTBE (3 × 15 mL), and after drying (MgSO_4_), the solvent was evaporated to leave a viscous residue (2.15 g). Crystallization was achieved by trituration with MTBE/heptane to yield **12**, as a colorless solid (1.67 g, 81%). TLC (heptane/MTBE (2:1)): R_f_ = 0.38. ^1^H-NMR (300 MHz, CDCl_3_) δ_H_ = 7.42 (dt, *J* = 7.3, 1.1 Hz, 1H), 7.38–7.35 (m, 1H), 7.31 (t, *J* = 7.7 Hz, 1H), 7.04 (ddd, *J* = 8.2, 2.8, 1.2, 30 Hz, 1H), 4.63 (d, *J* = 5.9 Hz, 2H), 4.45 (d, *J* = 5.9 Hz, 2H), 4.06 (s, 2H), 1.44 (s, 3H), 1.35 (s, 12H). ^13^C-NMR (75 MHz, CDCl_3_) δ_C_ = 158.65, 129.15, 127.59, 119.86, 118.27, 84.01, 80.01, 72.87, 39.86, 25.01, 21.47 (the boron-substituted carbon atom was not detectable).

*2-(2-Chloro-5-((3-methyloxetan-5 3-yl) methoxy) phenyl)-4,4,5,5-tetramethyl-1,3,2-dioxaborolane* (**9c**): *N*-Chlorosuccinimide (NCS, 0.4 g, 3.0 mmol) was added to a solution of compound **12** (0.73 g, 2.4 mmol) in DMF (7 mL). The mixture was stirred at room temperature for 20 h (monitored by TLC). The solvent was removed in vacuo, and the viscous residue was dissolved in MTBE (20 mL). The solution was successively washed with aqueous Na_2_S_2_O_3_ (10%, 9 mL) and water (6 mL) and extracted with aqueous NaOH (0.75 M, 3 × 6 mL). The alkaline extract was neutralized (→ pH 7–8) by slow addition of aqueous HCl (4 M) at 0–2 °C. The separated oil was extracted with MTBE (3 × 10 mL), and after drying (MgSO_4_), the solvent was evaporated to leave a viscous residue. Crystallization was achieved by trituration with MTBE/heptane to yield **9c**, as a colorless solid (0.45 g, 56%). TLC (heptane/MTBE (2:1)): R_f_ = 0.30. ^1^H-NMR (300 MHz, CDCl_3_) δ_H_ = 7.25 (d, *J* = 8.8 Hz, 1H), 7.23 (d, *J* = 3.2 Hz, 1H), 6.90 (dd, *J* = 8.8, 3.2 20 Hz, 1H), 4.61 (d, *J* = 5.9 Hz, 2H), 4.44 (d, *J* = 5.9 Hz, 2H), 4.01 (s, 2H), 1.42 (s, 3H), 1.37 (s, 12H). ^13^C-NMR (75 MHz, CDCl_3_) δ_C_ = 157.11, 131.36, 130.49, 121.90, 121.88, 118.33, 84.39, 79.90, 79.88, 73.15, 39.82, 24.95, 21.40.

#### 3.2.4. Synthesis of Compounds **BIT1**–**BIT9** via Suzuki Coupling According to General Procedure A

*1-(2-Chloro-5-methoxyphenyl)-8-(2-fluoropyridin-4-yl)-3methylbenzo[e]imidazo[5,1-c][1,2,4]triazine* (**BIT1**): Based on General Procedure A for Suzuki coupling, a mixture of compound **7a** (100 mg, 0.28 mmol, 1 eq), BA **9a** (63.4 mg,0.34 mmol, 1.2 eq), and K_2_CO_3_ (97 mg, 0.7 mmol, 2.5 eq) in dioxane/water (5 mL) was reacted in the presence of Pd(PPh_3_)_4_ (33 mg, 10 mol%) to give after purification by flash chromatography (gradient: hexane/ethyl acetate, 9:1 → ethyl acetate, 100%) compound **BIT1** (92 mg, 80%) as a yellow powder. TLC (hexane/ethyl acetate (1:1)): R_f_ = 0.33. ^1^H-NMR (400 MHz, CDCl_3_) δ_H_ = 8.55 (d, *J* = 8.4 Hz, 1H), 8.27 (d, *J* = 5.2 Hz, 1H), 7.85 (dd, *J* = 8.4, 1.7 Hz, 1H), 7.56 (d, *J* = 8.8 Hz, 1H), 7.48 (d, *J* = 1.6 Hz, 1H), 7.21 (d, *J* = 3.0 Hz, 1H), 7.19 (d, *J* = 3.1 Hz, 1H), 7.16 (d, *J* = 3.0 Hz, 1H), 6.88 (s, 1H), 3.87 (s, 3H), 2.99 (s, 3H). ^13^C-NMR (75 MHz, CDCl_3_) δ_C_ = 164.59 (d, *J* = 239.3 Hz), 159.09, 151.71 (d, *J* = 8.1 Hz), 148.68 (d, *J* = 15.4 Hz), 140.20 (d, *J* = 3.3 Hz), 139.84, 137.41, 136.98, 136.18, 131.51, 131.44, 130.81, 126.00, 124.05, 119.38 (d, *J* = 4.1 Hz), 118.56, 117.68, 113.90, 107.44 (d, *J* = 38.7 Hz), 56.01, 12.89. ^19^F-NMR (282 MHz, CDCl_3_) δ_F_ = −66.98 (s, br). HR-MS (ESI+): *m/z* = 420.1025 and 442.0848 (calcd. 420.1022 for C_22_H_16_ClFN_5_O**^+^** [M+H]^+^ and 442.0841 for C_22_H_15_ClFN_5_NaO**^+^** [M + Na]^+^).

*1-(2-Chloro-5-((3-methyloxetan-3-yl)methoxy)phenyl)-8-(6-fluoropyridin-3-yl)-3-methylbenzo[e]imidazo[5,1-c][1,2,4]triazine* (**BIT2**): According to General Procedure A, compound **7b** (144 mg, 0.40 mmol,1 eq), BA **9c** (163 mg, 0.48 mmol, 1.2 eq), K_2_CO_3_ (157 mg, 1.14 mmol, 2.84 eq), and Pd(PPh_3_)_4_ (23 mg, 5 mol%) were reacted in dioxane/water (5 mL) to give a raw product, which was purified by two-times column chromatography (CHCl_3_/MeOH (10:1) to yield compound **BIT2** (155 mg, 79%), as a yellow solid. TLC (hexane/ethyl acetate (1:3)): R_f_ = 0.29. ^1^H-NMR (400 MHz, CDCl_3_) δ_H_ = 8.52 (d, *J* = 8.3 Hz, 1H), 8.21 (d, *J* = 2.5 Hz, 1H), 7.90–7.81 (m, 1H), 7.77 (dd, *J* = 8.3, 1.8 Hz, 1H), 7.55 (d, *J* = 8.8 Hz, 1H), 7.45 (d, *J* = 1.8 Hz, 1H), 7.27 (s, 1H), 7.17 (dd, *J* = 8.9, 2.9 Hz, 1H), 7.02 (dd, *J* = 8.5, 3.0 Hz, 1H), 4.57 (dd, *J* = 6.0, 2.0 Hz, 2H), 4.44 (d, *J* = 5.9 Hz, 2H), 4.17–3.99 (m, 2H), 2.96 (s, 3H), 1.41 (s, 3H). ^13^C-NMR (101 MHz, CDCl_3_) δ_C_ = 163.85 (d, *J* = 242.3 Hz), 158.37, 146.27 (d, *J* = 15.2 Hz), 140.19, 139.79 (d, *J* = 8.1 Hz), 139.45, 137.37, 136.39, 135.92, 133.01 (d, *J* = 4.7 Hz), 131.74, 131.46, 130.85, 126.38, 125.94, 124.10, 118.73 (d, *J* = 41.2 Hz), 113.32, 110.38, 110.00, 79.66 (d, *J* = 3.4 Hz), 73.58, 39.77, 21.28, 12.87. ^19^F-NMR (282 MHz, CDCl_3_) δ_F_ = −67.82 (dd, *J* = 7.4, 3.2 Hz). HR-MS (ESI+): *m/z* = 490.1442 and 512.1260 (calcd. 490.1441 for C_26_H_22_ClFN_5_O_2_^+^ [M+H]^+^ and 512.1260 for C_26_H_21_ClFN_5_NaO_2_^+^ [M + Na]^+^).

*1-(2-Chloro-5-((3-methyloxetan-3-yl)methoxy)phenyl)-8-(2-fluoropyridin-4-yl)-3-methylbenzo[e]imidazo[5,1-c][1,2,4]triazine* (**BIT3**): According to General Procedure A, compound **7a** (100 mg, 0.279 mmol, 1 eq), BA **9c** (104 mg, 0.307 mmol, 1.1 eq), K_2_CO_3_ (93 mg, 0.642 mmol, 2.3 eq), and Pd(PPh_3_)_4_ (16 mg, 5 mol%) were reacted in dioxane/water (5 mL) to give after purification by flash chromatography (gradient: hexane/ethyl acetate, 1:2 → ethyl acetate, 100%) compound **BIT3** (93 mg, 68%), as a yellow solid. TLC (hexane/ethyl acetate (1:3)): R_f_ = 0.29. ^1^H-NMR (400 MHz, CDCl_3_) δ_H_ = 8.55 (d, *J* = 8.4 Hz, 1H), 8.27 (d, *J* = 5.2 Hz, 1H), 7.86 (dd, *J* = 8.4, 1.8 Hz, 1H), 7.58 (d, *J* = 8.9 Hz, 1H), 7.52 (d, *J* = 1.8 Hz, 1H), 7.28 (d, *J* = 3.0 Hz, 1H), 7.23–7.18 (m, 2H), 6.89 (d, *J* = 1.6 Hz, 1H), 4.60–4.55 (m, 2H), 4.44 (d, *J* = 6.0 Hz, 2H), 4.15–4.03 (m, 2H), 2.97 (s, 3H), 1.42 (s, 3H). ^13^C-NMR (101 MHz, CDCl_3_) δ_C_ = 164.60 (d, *J* = 239.3 Hz), 158.47, 151.79 (d, *J* = 8.2 Hz), 148.72 (d, *J* = 15.5 Hz), 140.25 (d, *J* = 3.4 Hz), 139.92, 137.45, 137.02, 136.10, 131.68, 131.49, 130.91, 126.37, 125.90, 124.06, 119.44 (d, *J* = 4.1 Hz), 118.91, 118.56, 114.00, 107.49 (d, *J* = 38.7 Hz), 79.63 (d, *J* = 4.1 Hz), 73.61, 39.77, 21.26, 12.90. ^19^F-NMR (282 MHz, CDCl_3_) δ_F_ = −66.93 (s, br). HR-MS (ESI+) *m/z* = 490.1443 and 512.1261 (calcd. 490.1441 for C**_26_**H**_22_**ClFN_5_O_2_^+^ [M + H]^+^ and 512.1260 for C**_26_**H**_21_**ClFN**_5_**NaO_2_^+^ [M + Na]^+^).

*1-(2-Chloro-5-methoxyphenyl)-8-(6-fluoropyridin-3-yl)-3 methylbenzo[e]imidazo[5,1-c][1,2,4]triazine* (**BIT4**): According to General Procedure A, compound **7b** (111 mg, 0.308 mmol, 1 eq), BA **9a** (69 mg, 0.370 mmol, 1.2 eq), K_2_CO_3_ (107 mg, 0.77 mmol, 2.5 eq), and Pd(PPh_3_)_4_ (36 mg, 10 mol%) were reacted in dioxane/water (5 mL) to give after purification by flash chromatography (gradient: CHCl_3_/MeOH, 50:1 → CHCl_3_/MeOH, 30:1) compound **BIT4** (83 mg, 64%), as a yellow solid. TLC (hexane/ethyl acetate (1:1)): R_f_ = 0.37. ^1^H-NMR (400 MHz, CDCl_3_) δ_H_ = 8.51 (d, *J* = 8.4 Hz, 1H), 8.20 (d, *J* = 2.3 Hz, 1H), 7.82 (ddd, *J* = 8.6, 7.3, 2.7 Hz, 1H), 7.76 (dd, *J* = 8.4, 1.8 Hz, 1H), 7.52 (d, *J* = 8.9 Hz, 1H), 7.39 (d, *J* = 1.8 Hz, 1H), 7.19 (d, *J* = 3.1 Hz, 1H), 7.13 (dd, *J* = 8.9, 3.0 Hz, 1H), 7.01 (dd, *J* = 8.5, 3.1 Hz, 1H), 3.84 (s, 3H), 2.96 (s, 3H).^13^C-NMR (75 MHz, CDCl_3_) δ_C_ = 163.82 (d, *J* = 242.0 Hz), 159.00, 146.24 (d, *J* = 15.0 Hz), 140.19, 139.76 (d, *J* = 8.1 Hz), 139.38, 137.33, 136.36, 136.00, 132.98 (d, *J* = 4.7 Hz), 131.58, 131.43, 130.77, 126.04, 125.87, 124.10, 118.64, 117.62, 113.26, 110.16 (d, *J* = 37.5 Hz), 55.99, 12.86. ^19^F-NMR (282 MHz, CDCl_3_) δ_F_ = −67.90 (dd, *J* = 7.4, 3.1 Hz). HR-MS (ESI+): *m/z* = 420.1010 (calcd. 420.1022 for C_22_H_16_ClFN_5_O^+^ [M + H]^+^).

*1-(2-Chloro-5-methoxyphenyl)-8-(2-fluoropyridin-3-yl)-3 methylbenzo[e]imidazo[5,1-c][1,2,4]triazine* (**BIT5**): According to General Procedure A, compound **7c** (115 mg, 0.322 mmol, 1 eq), BA **9a** (72 mg, 0.386 mmol, 1.2 eq), K_2_CO_3_ (115 mg, 0.832 mmol, 2.58 eq), and Pd(PPh_3_)_4_ (37 mg, 10 mol%) were reacted in dioxane/water (5 mL) to give after purification by flash chromatography (gradient: light petroleum ether/ethyl acetate, 1:1 → light petroleum ether/ethyl acetate, 1:2) compound **BIT5** (93 mg, 69%), as a yellow solid. TLC (light petroleum ether/ethyl acetate (1:2)): R_f_ = 0.43. ^1^H-NMR (300 MHz, CDCl_3_) δ_H_ = 8.51 (d, *J* = 8.4 Hz, 1H), 8.22 (dd, *J* = 4.8, 0.7 Hz, 1H), 7.83–7.73 (m, 2H), 7.52–7.45 (m, 2H), 7.32–7.27 (m, 1H), 7.17 (d, *J* = 3.0 Hz, 1H), 7.10 (dd, *J* = 8.9, 3.0 Hz, 1H), 3.83 (s, 3H), 2.97 (s, 3H). ^13^C-NMR (75 MHz, CDCl_3_) δ_C_ = 160.19 (d, *J* = 242.0 Hz), 158.95, 147.84 (d, *J* = 15.0 Hz), 140.67 (d, *J* = 3.5 Hz), 139.44, 137.51, 137.42 (d, *J* = 2.1 Hz), 136.37, 136.21, 131.33, 131.01, 130.93, 127.58 (d, *J* = 2.8 Hz), 126.05, 123.76, 122.28 (d, *J* = 4.6 Hz), 121.95 (d, *J* = 27.2 Hz), 118.72, 117.03, 115.51 (d, *J* = 5.2 Hz), 55.95, 12.88. ^19^F-NMR (282 MHz, CDCl_3_) δ_F_ = −70.11 (d, *J* = 9.8 Hz). HR-MS (ESI+): *m/z* = 420.1014 (calcd. 420.1022 for C_22_H_16_ClFN_5_O^+^ [M + H]^+^).

*1-(2-Fluoro-5-methoxyphenyl)-8-(2-fluoropyridin-4-yl)-3-methylbenzo[e]imidazo[5,1-c][1,2,4]triazine* (**BIT6**): According to General Procedure A, compound **7a** (100 mg, 0.279 mmol, 1 eq), BA **9b** (62 mg, 0.365 mmol, 1.3 eq), K_2_CO_3_ (97 mg, 0.698 mmol, 2.58 eq), and Pd(PPh_3_)_4_ (32 mg, 10 mol%) were reacted in dioxane/water (5 mL) to give after purification by flash chromatography (gradient: light petroleum ether/ethyl acetate, 1:1 → light petroleum ether/ethyl acetate, 1:2) compound **BIT6** (81 mg, 72%), as an orange solid. TLC (petroleum ether/ethyl acetate (1:2)): R_f_ = 0.54. ^1^H-NMR (300 MHz, CDCl_3_) δ_H_ = 8.55 (d, *J* = 8.4 Hz, 1H), 8.27 (dd, *J* = 5.3, 0.7 Hz, 1H), 7.86 (dd, *J* = 8.4, 1.9 Hz, 1H), 7.73 (dd, *J* = 3.5, 1.8 Hz, 1H), 7.30–7.27 (m, 1H), 7.25–7.14 (m, 3H), 6.94–6.91 (m, 1H), 3.88 (s, 3H), 2.97 (s, 3H).^13^C-NMR (75 MHz, CDCl_3_) δ_C_ = 164.61 (d, *J* = 239.5 Hz), 156.62 (d, *J* = 7.1 Hz), 153.86 (d, *J* = 246.3 Hz), 153.58, 151.80, 148.73 (d, *J* = 15.4 Hz), 140.32, 140.24 (d, *J* = 2.9 Hz), 137.50 (d, *J* = 62.8 Hz), 133.85, 131.56, 125.94, 124.28, 120.00 (d, *J* = 16.3 Hz), 119.46 (d, *J* = 4.1 Hz), 118.76 (d, *J* = 7.9 Hz), 117.03 (d, *J* = 22.7 Hz), 116.44, 113.90 (d, *J* = 2.2 Hz), 107.56 (d, *J* = 38.8 Hz), 56.21, 12.88.^19^F-NMR (282 MHz, CDCl_3_) δ_F_ = −66.90––66.97 (m), −121.68–121.78 (m). HR-MS (ESI+): *m/z* = 404.1325 (calcd. 404.1317 for C_22_H_16_F_2_N_5_O**^+^** [M + H]^+^).

*1-(2-Chloro-5-methoxyphenyl)-8-(3-fluoropyridin-5-yl)-3 methylbenzo[e]imidazo[5,1-c][1,2,4]triazine* (**BIT7**): According to General Procedure A, compound **7d** (100 mg, 0.279 mmol, 1 eq), BA **9a** (62 mg, 0.335 mmol, 1.2 eq), K_2_CO_3_ (116 mg, 0.839 mmol, 3.0 eq), and Pd(PPh_3_)_4_ (17 mg, 5 mol%) were reacted in dioxane/water (5 mL) to give after purification by flash chromatography (gradient: hexane/ethyl acetate, 1:2 → hexane/ethyl acetate, 1:3) compound **BIT7** (79 mg, 67**%**), as a yellow solid. TLC (hexane/ethyl acetate (1:2)): R_f_ = 0.38. ^1^H-NMR (400 MHz, CDCl_3_) δ_H_ = 8.53 (d, *J* = 8.4 Hz, 1H), 8.48 (d, *J* = 2.7 Hz, 1H), 8.46–8.45 (m, 1H), 7.81 (dd, *J* = 8.4, 1.9 Hz, 1H), 7.55 (d, *J* = 8.9 Hz, 1H), 7.44–7.38 (m, 2H), 7.20 (d, *J* = 3.0 Hz, 1H), 7.14 (dd, *J* = 8.9, 3.0 Hz, 1H), 3.85 (s, 3H), 2.97 (s, 3H). ^13^C-NMR (101 MHz, CDCl_3_) δ_C_ = 159.71 (d, *J* = 258.4 Hz), 159.05, 143.99 (d, *J* = 4.0 Hz), 139.77, 139.56, 138.21 (d, *J* = 23.1 Hz), 137.37, 136.58, 136.23 (d, *J* = 3.8 Hz), 136.09, 131.52, 131.48, 130.84, 126.02, 125.99, 124.10, 121.21 (d, *J* = 19.0 Hz), 118.59, 117.70, 113.68, 55.99, 12.87. ^19^F-NMR (377 MHz, CDCl_3_) δ_F_ = −125.95 (d, *J* = 9.2 Hz). HR-MS (ESI+): *m/z* = 420.1024 (calcd. 420.1022 for C_22_H_16_ClFN_5_O**^+^** [M + H]^+^).

*1-(2-Fluoro-5-methoxyphenyl)-3-methyl-8-(pyridin-3-yl)benzo[e]imidazo[5,1-c][1,2,4]triazine* (**BIT8**): According to General Procedure A, compound **7e** (100 mg, 0.294 mmol, 1 eq), BA **9b** (60 mg, 0.353 mmol, 1.2 eq), K_2_CO_3_ (110 mg, 0.796 mmol, 2.7 eq), and Pd(PPh_3_)_4_ (17 mg, 5 mol%) were reacted in dioxane/water (5 mL) to give after purification by flash chromatography (gradient: hexane/ethyl acetate, 1:3 → ethyl acetate, 100%) compound **BIT8** (93 mg, 82**%**), as an orange solid. TLC (hexane/ethyl acetate (1:3)): R_f_ = 0.35. ^1^H-NMR (300 MHz, CDCl_3_) δ_H_ = 8.69–8.62 (m, 2H), 8.53 (d, *J* = 8.4 Hz, 1H), 7.83 (dd, *J* = 8.4, 1.8 Hz, 1H), 7.77 (ddd, *J* = 8.0, 2.4, 1.6 Hz, 1H), 7.70 (dd, *J* = 3.5, 1.8 Hz, 1H), 7.39 (ddd, *J* = 8.0, 4.8, 0.8 Hz, 1H), 7.30–7.23 (m, 2H), 7.18–7.11 (m, 1H), 3.86 (s, 3H), 2.97 (s, 3H). ^13^C-NMR (75 MHz, CDCl_3_) δ_C_ = 156.52 (d, *J* = 1.8 Hz), 154.85 (d, *J* = 243.0 Hz), 149.93, 148.39, 140.58 (d, *J* = 125.3 Hz), 137.76, 136.60, 134.74, 134.53, 133.64, 131.43, 128.62 (d, *J* = 12.4 Hz), 126.12, 124.28, 123.94, 120.12 (d, *J* = 16.6 Hz), 118.83 (d, *J* = 7.9 Hz), 117.01 (d, *J* = 22.9 Hz), 116.29 (d, *J* = 2.0 Hz), 113.35 (d, *J* = 1.9 Hz), 56.18, 12.86. ^19^F-NMR (282 MHz, CDCl_3_) δ_F_ = −121.71 (td, *J* = 9.0, 4.0 Hz). HR-MS (ESI+): *m/z* = 386.1400 (calcd. 386.1412 for C_22_H_17_FN_5_O^+^ [M + H]^+^).

*1-(2-Fluoro-5-methoxyphenyl)-3-methyl-8-(pyridin-4-yl)benzo[e]imidazo[5,1-c][1,2,4]triazine* (**BIT9**): According to General Procedure A, compound **7f** (100 mg, 0.294 mmol, 1 eq), BA **9b** (61 mg, 0.366 mmol, 1.2 eq), K_2_CO_3_ (103 mg, 0.745 mmol, 2.53 eq), and Pd(PPh_3_)_4_ (19 mg, 5 mol%) were reacted in dioxane/water (5 mL) to give a raw product, which was purified by two-times flash chromatography: CHCl_3_/MeOH (10:1) was used first, followed by hexane/ethyl acetate (gradient 1:3 → ethyl acetate, 100%) to obtain compound **BIT9** (44 mg, 39**%**), as a yellow solid. TLC (hexane/ethyl acetate (1:1)): R_f_ = 0.29. ^1^H-NMR (300 MHz, CDCl_3_) δ_H_ = 8.68–8.65 (m, 2H), 8.53 (d, *J* = 8.5 Hz, 1H), 7.87 (dd, *J* = 8.4, 1.8 Hz, 1H), 7.74 (ddd, *J* = 3.5, 1.9, 0.5 Hz, 1H), 7.33–7.30 (m, 2H), 7.29–7.22 (m, 2H), 7.19–7.13 (m, 1H), 3.87 (s, 3H), 2.96 (s, 3H). ^13^C-NMR (101 MHz, CDCl_3_) δ_C_ = 156.57 (d, *J* = 2.0 Hz), 154.85 (d, *J* = 243.4 Hz), 150.60, 146.38, 141.51, 140.07, 137.81, 137.04, 133.73, 131.46, 125.98, 124.28, 121.65, 120.11 (d, *J* = 16.6 Hz), 118.86 (d, *J* = 7.9 Hz), 117.01 (d, *J* = 22.6 Hz), 116.24 (d, *J* = 2.0 Hz), 113.67 (d, *J* = 2.3 Hz), 56.21, 12.87. ^19^F-NMR (282 MHz, CDCl_3_) δ_F_ = −121.63–-121.71 (m). HR-MS (ESI+): *m/z* = 386.1404 (calcd. 386.1412 for C_22_H_17_FN_5_O**^+^** [M + H]^+^).

### 3.3. Biology

#### 3.3.1. In Vitro Evaluation of BIT Derivatives Towards PDEs

The inhibitory potency of BIT derivatives against the human recombinant PDE subtype was determined in enzyme assays, conducted by SB Drug Discovery (Scotland, U.K.). The phosphodiesterase assays were performed using recombinant human PDE enzymes expressed in a baculoviral system. This had similarity to PDE enzymes taken from human tissue using known inhibitor standards where available. The radiometric assay was a modification of the two-step method of Thompson and Appleman [45], which was adapted for 96-well plate format.

Firstly, tritium-labeled cyclic AMP or cyclic GMP was hydrolyzed to 5′-AMP or 5′-GMP by phosphodiesterase. The 5′-AMP or 5′-GMP was then further hydrolyzed to adenosine or guanosine by nucleotidase in snake venom. An anion-exchange resin bound all charged nucleotides and left [^3^H]adenosine or [^3^H]guanosine as the only labeled compound to be counted by liquid scintillation. Briefly, 50 µL of diluted human PDE enzyme were incubated with 50 µL of [^3^H]-cAMP or [^3^H]-cGMP and 11 µL of 50% DMSO (or compound dilution) for 20 min (at 30 °C). Reactions were carried out in a Greiner 96-deep well 1-mL master-block. The enzyme was diluted in 20 mM Tris HCl pH 7.4 and [^3^H]-cAMP or [^3^H]-cGMP are diluted in 10 mM MgCl_2_, 40 mM Tris HCl pH 7.4. The reaction is terminated by denaturing the PDE enzyme (at 70 °C for 2 min), after which 25 µL of snake venom nucleotidase were added and incubated for 10 min (at 30 °C). Plates were centrifuged for 9 seconds before incubation. After incubation, 200 µL of Dowex resin were added, and the plate was shaken for 20 min then centrifuged for 3 min at 2500 r.p.m. Fifty microliters of supernatant were removed and added to 200 µL of MicroScint-20 in white plates (Greiner 96-well Optiplate) and shaken for 30 min before reading on a Perkin Elmer TopCount Scintillation Counter.

Compounds were tested at a concentration of 10 nM against human PDE2A3 and at 1 µM for PDE4A1, PDE5A, PDE6AB, PDE9A1, and PDE10A1. The percentage of inhibition of the compounds and standard inhibitors was determined and compared to historical assay data to ensure that they fell within acceptable ranges. For IC_50_ values’ measurements, **BIT1** was tested at concentrations of 0.1, 0.5, 1.0, 5.0, 10.0, and 100 nM, and **BIT6** and **BIT9** were tested at concentrations of 0.5, 1.0, 5.0, 10.0, 100, and 1000 nM against PDE2A3, while the concentration against PDE10A1 at 0.25, 5.0, 10, 100, 250, and 1000 nM (*n* = 2).

Data generated were analyzed using Prism software (GraphPad Inc.).

#### 3.3.2. Incubations with Mouse Liver Microsomes 

For microsome experiments, the following instruments were used: BioShake iQ (QUANTIFOIL Instruments, Jena, Germany), Centrifuge 5424 (Eppendorf, Hamburg, Germany), DB-3D TECHNE Sample Concentrator (Biostep, Jahnsdorf, Germany), and UltiMate 3000 UHPLC System (Thermo Scientific, Germering, Germany) including a DAD detector (DAD-3000RS) coupled to an MSQ Plus Single Quadrupole Mass Spectrometer (Thermo Scientific, Austin, Texas, USA).

NADPH (Nicotinamide Adenine Dinucleotide Phosphate) and testosterone were purchased from Sigma-Aldrich (Steinheim, Germany). GIBCO Mouse Liver Microsomes (MLM, 20 mg/mL) were purchased from Life Technologies (Darmstadt, Germany). Dulbecco’s Phosphate-Buffered Saline (PBS) (without Ca^2+^, Mg^2+^) was purchased from Biochrom (Berlin, Germany). 

Incubations had a final volume of 250 µL and were performed in PBS (pH 7.4). **BIT1** was freshly dissolved in DMSO to provide a stock solution of 2 mM that was used for the experiments and resulted in an amount of DMSO of 1% in each incubation mixture. In the following, final concentrations are provided in brackets [43]. PBS, MLM (1 mg/mL), and **BIT1** (20 µM) were mixed and preincubated at 37 °C for 5 min. Analogously, preincubated NADPH (2 mM) was added, and the mixtures were gently shaken at 37 °C. After 90 min, portions of 1.0 mL of cold acetonitrile (−20 °C) were added, respectively, followed by vigorous shaking (30 s), cooling on ice (4 min), and centrifugation at 14,000 rpm (10 min). Supernatants were concentrated at 50 °C under a flow of nitrogen to provide residual volumes of 40–60 µL, which were reconditioned by adding acetonitrile/water 1:1 (*v*/*v*) to obtain samples of 100 µL, which were stored at 4 °C until analyzed by HPLC-UV-MS. As a positive control, testosterone was used as the substrate and incubated at an appropriate concentration, similar to the protocol described above, to give complete conversion confirmed by HPLC. Furthermore, incubations without NADPH, microsomal protein, and **BIT1**, respectively, were performed as negative controls.

HPLC-UV-MS analyses were performed on a ReproSil-Pur 120 C18-AQ-column, 125 mm × 3 mm, 3 µm (Dr. Maisch GmbH, Ammerbuch, Germany) equipped with an appropriate precolumn at 25 °C and a flow rate of 0.7 mL/min. The solvent system consisted of Eluent A: water, containing 2 mM ammonium acetate, and Eluent B: water/acetonitrile 20/80 (v/v), containing 2 mM ammonium acetate. Two methods were applied. Method A (gradient elution, % acetonitrile): 0–1.5 min, 10%; 1.5–10 min, 10–80%; 10-13 min, 80%; 13–16 min 10%. Method B (isocratic elution, % acetonitrile): 0–25 min, 42%. For both methods, UV detection was performed at a wavelength of 228 nm (maximum UV absorbance of **BIT1**) and a bandwidth of 20 nm. MSQ Plus single quadrupole mass spectrometer was operated in positive electrospray ionization mode: probe temperature 500 °C, needle voltage 5 V, cone voltage 75 V.

## 4. Conclusions

A series of novel fluorinated PDE2A inhibitors based on a **BIT** (benzoimidazotriazine) scaffold was successfully prepared and evaluated in vitro. The binding results revealed that a modification with a 2,2-oxetanyl propoxy portion on the phenyl (phe-3) at the 1-position led to a potency loss towards PDE2A. However, a small methoxy group at the same position (phe-1) led to a significant increase of potency towards PDE2A. It appeared that a higher inhibition towards PDE2A was obtained by pyridine-4-yl residues in this position, either fluorinated or non-fluorinated. Furthermore, the introduction of ortho-fluorophenyl at the 1-position, instead of o-chlorophenyl, showed a lack of selectivity towards PDE2A. The introduction of the 2-fluoropyridin-4-yl residue at the 8-position provided us a promising candidate for future labeling with fluorine-18.

In vitro metabolism studies of **BIT1** with MLM showed that **BIT1** was sufficiently stable and suitable for the development of an ^18^F-labeled radioligand. This should further be proven by in vivo investigations in the future. Taken together, **BIT1** might be a prospective ligand to be developed as a radioligand for PDE2A-PET imaging. 

## Figures and Tables

**Table 1:** Yields of Suzuki coupling with compound **5** (products: **6a**–**6f**) and of bromination (products: **7a**–**7f**).

Entry	Starting Boronic Acid (BA)	Product of First Suzuki Coupling	Yield	Product of Bromination	Yield
1	2-fluoro-pyridine-4-yl-BA (**8a**)	**6a**	76%	**7a**	79%
2	6-fluoro-pyridine-3-yl-BA (**8b**)	**6b**	28%	**7b**	63%
3	2-fluoro-pyridine-3-yl-BA (**8c**)	**6c**	98%	**7c**	88%
4	3-fluoro-pyridine-5-yl-BA (**8d**)	**6d**	56%	**7d**	98%
5	pyridine-3-yl-BA (**8e**)	**6e**	77%	**7e**	87%
6	pyridine-4-yl-BA (**8f**)	**6f**	87%	**7f**	92%

**Table 2:** Series of BIT derivatives according to Scheme 2.

			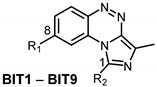		
Entry	Bromo Compound	Boronic Acid Derivative	R_1_/R_2_	Product	Yield
1	**7a**	**9a**	2-F-pyr-4/phe-1	**BIT1**	80%
2	**7b**	**9c**	6-F-pyr-3/phe-3	**BIT2**	79%
3	**7a**	**9c**	2-F-pyr-4/phe-3	**BIT3**	68%
4	**7b**	**9a**	6-F-pyr-3/phe-1	**BIT4**	64%
5	**7c**	**9a**	2-F-pyr-3/phe-1	**BIT5**	69%
6	**7a**	**9b**	2-F-pyr-4/phe-2	**BIT6**	72%
7	**7d**	**9a**	5-F-pyr-3/phe-1	**BIT7**	56%
8	**7e**	**9b**	pyr-3/phe-2	**BIT8**	82%
9	**7f**	**9b**	pyr-4/phe-2	**BIT9**	39%

**Table 3:** Percentage inhibition of PDE sub-types from synthesized compounds; for PDE2A3, the compounds were measured at a concentration of 10 nM and for PDE4A1, PDE5A1, PDE9A1, and PDE10A1 at 1 µM.

	Inhibition of PDE Sub-Types (%)				
	2A3	4A1	5A1	9A1	10A1
**BIT1**	82.9	58.6	29.3	NI	32.4
**BIT2**	8.52	64.1	NI	NI	2.46
**BIT3**	13.2	72.7	NI	NI	28.2
**BIT4**	2.63	NI	NI	NI	10.8
**BIT5**	NI	NI	NI	NI	22.1
**BIT6**	56.6	54.2	13.6	28.4	74.0
**BIT7**	50.6	65.7	25.1	11.5	87.0
**BIT8**	NI	13.9	36.2	NI	55.5
**BIT9**	80.5	86.8	57.7	17.5	96.6
**TA1**	93.5	24.9	3.08	NI	41
NI: No Inhibition					

**Table 4:** Affinity and selectivity of three new fluorinated compounds towards PDE2A and PDE10A.

Compounds	IC_50_ PDE2A (nM)	IC_50_ PDE10A (nM)	Selectivity Ratio
**BIT1**	3.33	53.23	16
**BIT6**	65.06	168.4	2.5
**BIT9**	17.7	20.24	1.1

**Table 5:** Percentage inhibition of PDE sub-types by **BIT1** (measured at 1 µM).

PDE Subtypes	%Inhibition
PDE1A3	12.6
PDE3A	20
PDE6AB	16.8
PDE7A	16.4
PDE8A1	23.3
PDE11A	14.5

**Table 6:** Overview of HPLC and MS data of in vitro metabolites of **BIT1** formed by MLM in the presence of NADPH.

Metabolite	t_R_ (min) Method A Gradient	t_R_ (min) Method B Isocratic	*m/z* Found	*m/z* Theoret.	Identification
***BIT1***	*11.28*	*16.10*	*420.7*	*420.1*	*parent (M + H)^+^*
**M1**	8.47	2.85	422.7	422.1	reduction (M + 2H + H)^+^
**M2**	9.05	4.24	452.8	452.1	di-oxygenation (M + 2O + H)^+^
**M3**	9.33	5.02	436.7	436.1	mono-oxygenation (M + O + H)^+^
**M4**	9.86	7.03	406.6	406.1	demethylation (M-CH_3_ + H)^+^
**M5**	10.67	12.04	436.7	436.1	mono-oxygenation (M + O + H)^+^
**M6**	not detected	17.89	436.7	436.1	mono-oxygenation (M + O + H)^+^

## Supplementary Materials

Supplementary Materials including NMR spectra of final compounds **BIT1**–**BIT9**, and dose response curves (**BIT1**, **BIT6**, and **BIT9**) can be accessed at.
