# Supplementary material for: Synthesis and In Vitro Evaluation of 8-Pyridinyl-Substituted Benzo[e]imidazo[2,1-c][1,2,4]triazines as Phosphodiesterase 2A Inhibitors
Source: Molecules. 2019 Jul 31;24(15):2791. doi: 10.3390/molecules24152791 (PMC6696243; doi:10.3390/molecules24152791)
Supplement: Supplementary file 1 [file molecules-24-02791-s001.pdf]

molecules

MDPI

Supplementary Information

Synthesis and In Vitro Evaluation of 8-Pyridinyl-Substituted Benzo[*e*]imidazo[2,1-*c*][1,2,4]triazines as Phosphodiesterase 2A Inhibitors

Rien Ritawidya [1,2,*], Friedrich-Alexander Ludwig [1], Detlef Briel [3], Peter Brust [1] and Matthias Scheunemann [1,*]

[1] Department of Neuroradiopharmaceuticals, Institute of Radiopharmaceuticals Cancer Research, Helmholtz-Zentrum Dresden-Rossendorf, Leipzig 04318, Germany

[2] Center for Radioisotope and Radiopharmaceutical Technology, National Nuclear and Energy Agency (BATAN), Puspiptek Area, Serpong, South Tangerang, Indonesia

[3] Pharmaceutical/Medicinal Chemistry, Institute of Pharmacy, Faculty of Medicine, Leipzig University, Brüderstraße 34, Leipzig 04103, Germany

* Correspondence: r.ritawidya@hzdr.de or rienrita@batan.go.id; m.scheunemann@hzdr.de (M.S.); Tel.: +49-341-234-179-4611 (R.R.); +49-341-234-179-4618 (M.S.)

Table of Contents

Images oF-NMR spectrum and HRMS (ESI) of final compounds

Figure S1. A. ^{1}H-NMR (400 MHz, $CDCl_3$), **B**. ^{13}C-NMR (75 MHz, $CDCl_3$) spectrum of **BIT1** **S3**

Figure S2. A. ^{19}F-NMR (282 MHz, $CDCl_3$), **B**. HRMS (ESI+) spectrum of **BIT1** **S4**

Figure S3. A. ^{1}H-NMR (400 MHz, $CDCl_3$), **B**. ^{13}C-NMR (101 MHz,$CDCl_3$) spectrum of **BIT2** **S5**

Figure S4. A. ^{19}F-NMR (282 MHz, $CDCl_3$) ,**B**. HRMS (ESI+) spectrum, of **BIT2** **S6**

Figure S5. A. ^{1}H-NMR (400 MHz, $CDCl_3$), **B**. ^{13}C-NMR (101 MHz, $CDCl_3$) spectrum of **BIT3** **S7**

Figure S6. A. ^{19}F-NMR (282 MHz, $CDCl_3$), **B**. HRMS (ESI+) spectrum of **BIT3** **S8**

Figure S7. A. ^{1}H-NMR (400 MHz, $CDCl_3$), **B**. ^{13}C-NMR (75 MHz, $CDCl_3$) spectrum of **BIT4** **S9**

Figure S8. A. ^{19}F-NMR (282 MHz, $CDCl_3$), **B**. HRMS (ESI+) spectrum of **BIT4** **S10**

Figure S9. A. ^{1}H-NMR (300 MHz, $CDCl_3$), **B**. ^{13}C-NMR (75 MHz, $CDCl_3$) spectrum of **BIT5** **S11**

Figure S10. A. ^{19}F-NMR (282 MHz, $CDCl_3$), **B**. HRMS (ESI+) spectrum of **BIT5** **S12**

Figure S11. A. ^{1}H-NMR (300 MHz, $CDCl_3$), **B**. ^{13}C-NMR (75 MHz, $CDCl_3$) spectrum of **BIT6** **S13**

Figure S12. A. ^{19}F-NMR (282 MHz, $CDCl_3$), **B**. HRMS (ESI+) spectrum of **BIT6** **S14**

Figure S13. A. ^{1}H-NMR (400 MHz, $CDCl_3$), **B**. ^{13}C-NMR (101 MHz,$CDCl_3$) spectrum of **BIT7** **S15**

Figure S14. A. ^{19}F-NMR (377 MHz, $CDCl_3$), **B**. HRMS (ESI+) spectrum of **BIT7** **S16**

Figure S15. A. ^{1}H-NMR (300 MHz, $CDCl_3$), **B**. ^{13}C-NMR (75 MHz, $CDCl_3$) spectrum of **BIT8** **S17**

Figure S16. A. ^{19}F-NMR (282 MHz, $CDCl_3$), **B**. HRMS (ESI+) spectrum of **BIT8** **S18**

Figure S17. A. ^{1}H-NMR (300 MHz, $CDCl_3$), **B**. ^{13}C-NMR (101 MHz, $CDCl_3$) spectrum of **BIT9** **S19**

Figure S18. A. ^{19}F-NMR (282 MHz, $CDCl_3$), **B**. HRMS (ESI+) spectrum of **BIT9** **S20**

2. In vitro evaluation (Dose Response Curve) (SB Drug Discovery-Scottland)

Figure S19. A. BIT1, **BIT6**, **BIT9** towards PDE2A and PDE10A, **B. BAY60-7550** and **TA1** towards PDE2A **S21**

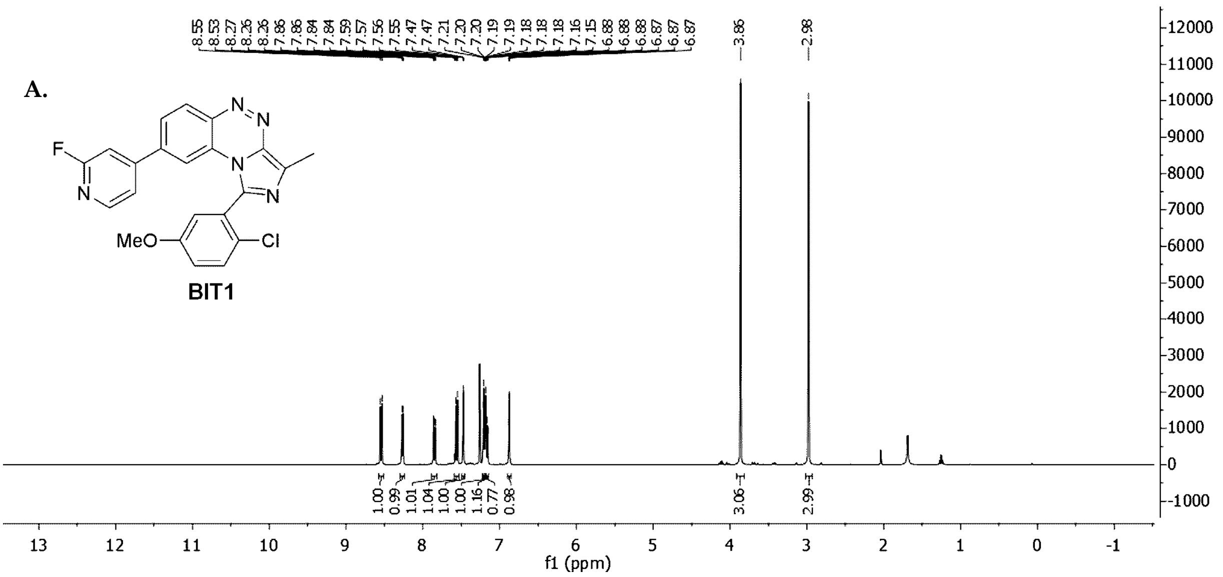

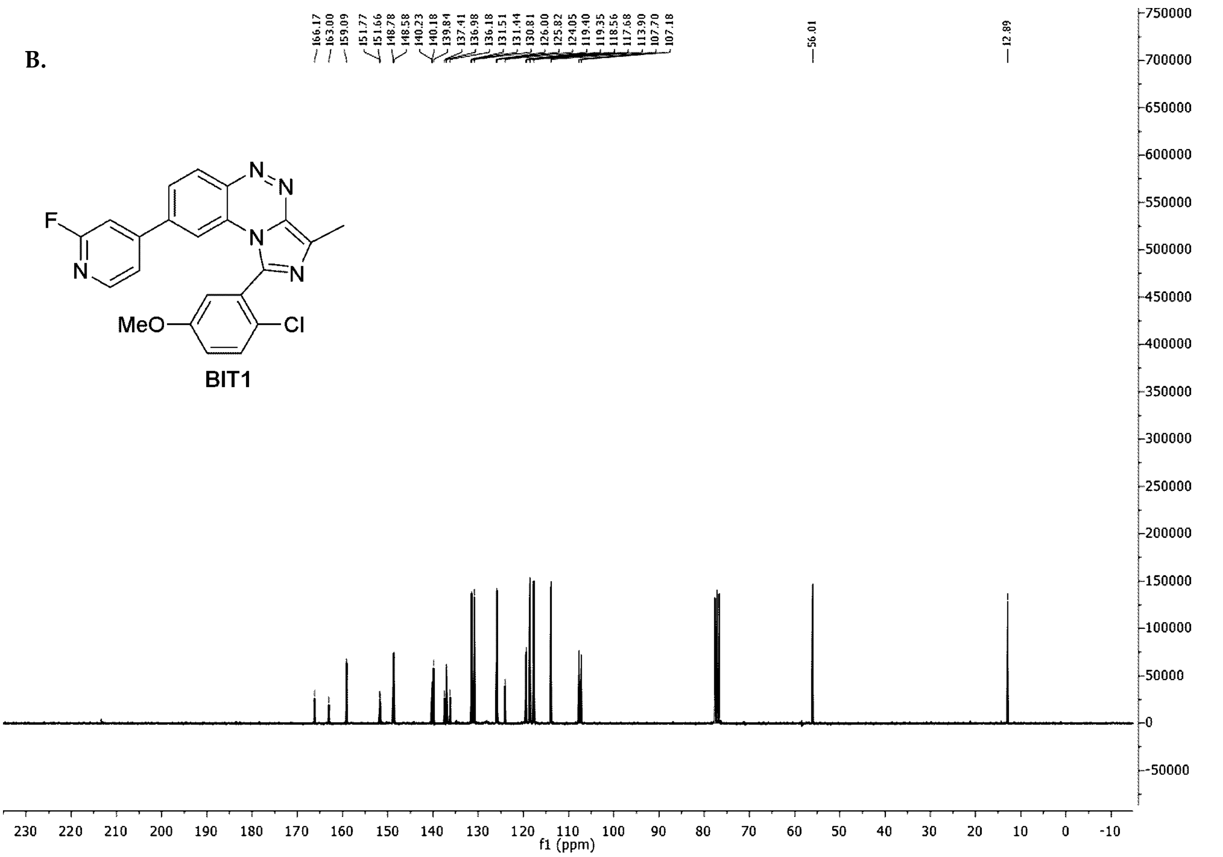

Figure S1. A. ^{1}H-NMR and **B**. ^{13}C-NMR spectrum of **BIT1** in $CDCl_3$

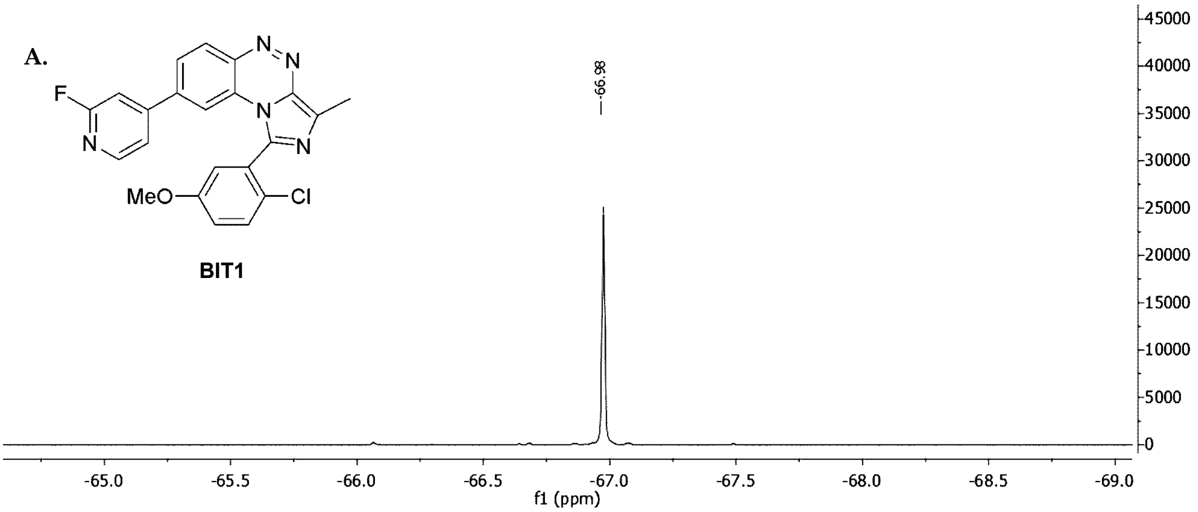

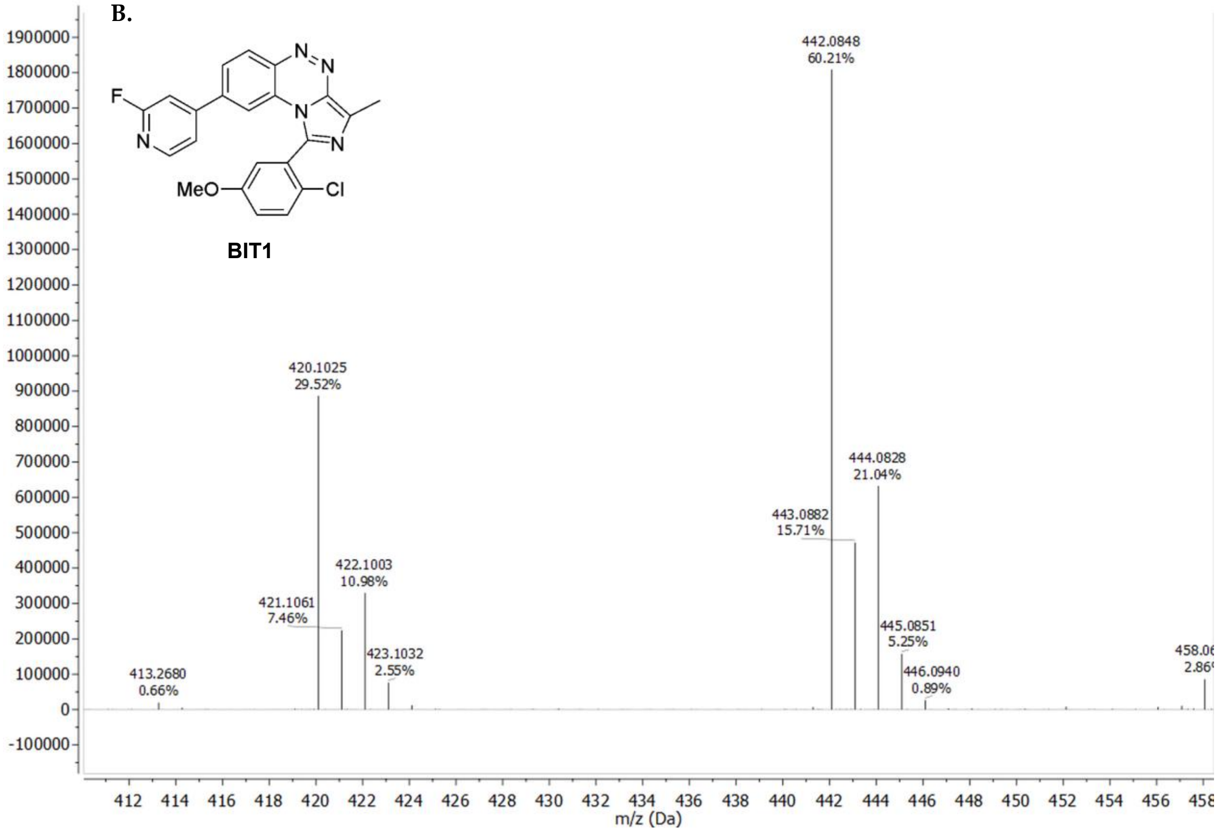

Figure S2. A. ^{19}F-NMR spectrum of **BIT1** in $CDCl_3$ and **B**.HRMS (ESI+) spectrum of **BIT1**

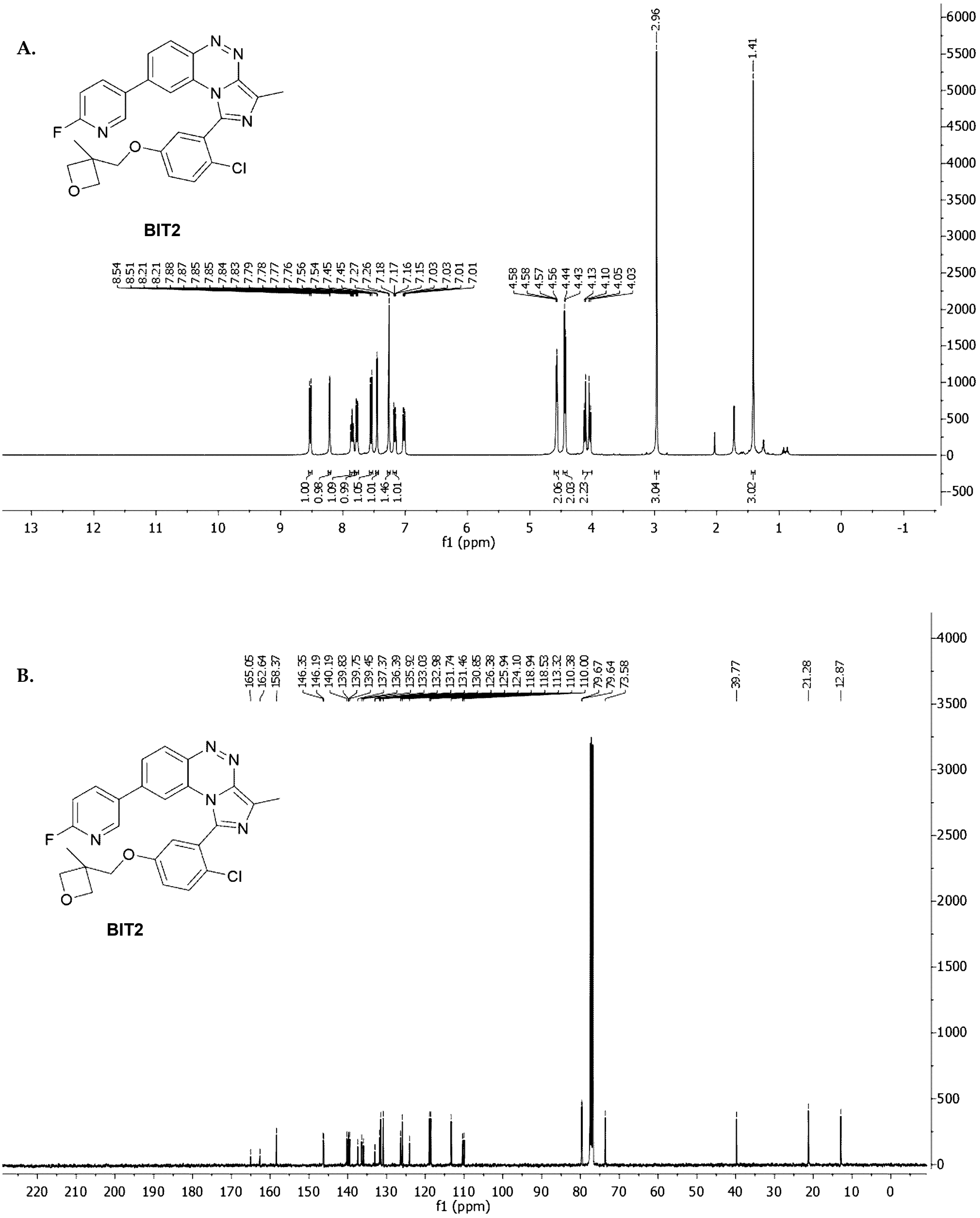

Figure S3. A. ^{1}H-NMR and **B.** ^{13}C-NMR spectrum of **BIT2** in $CDCl_3$

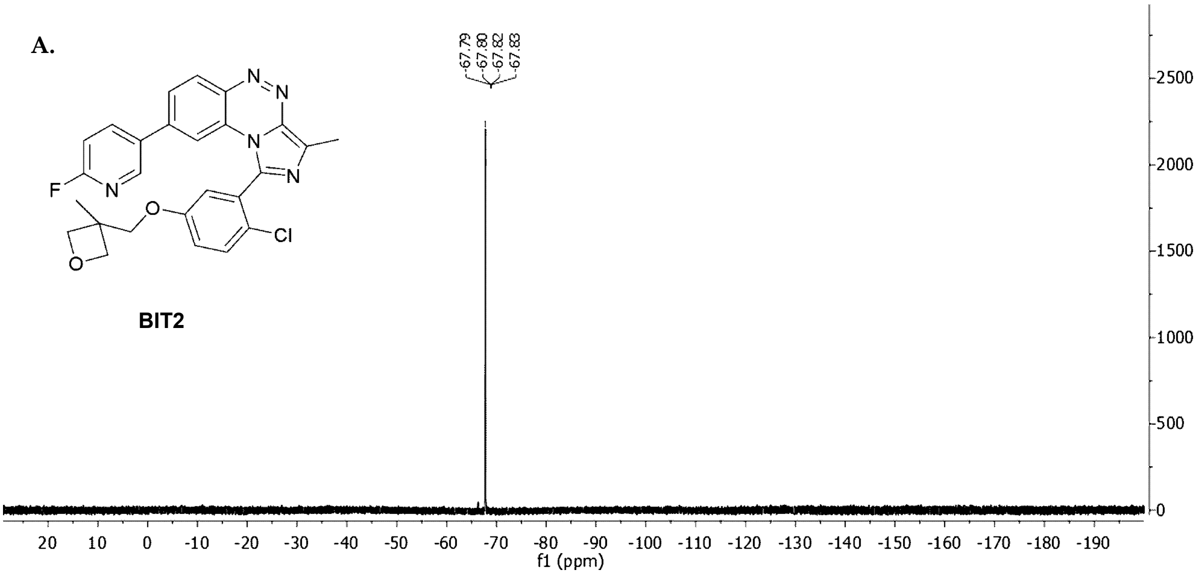

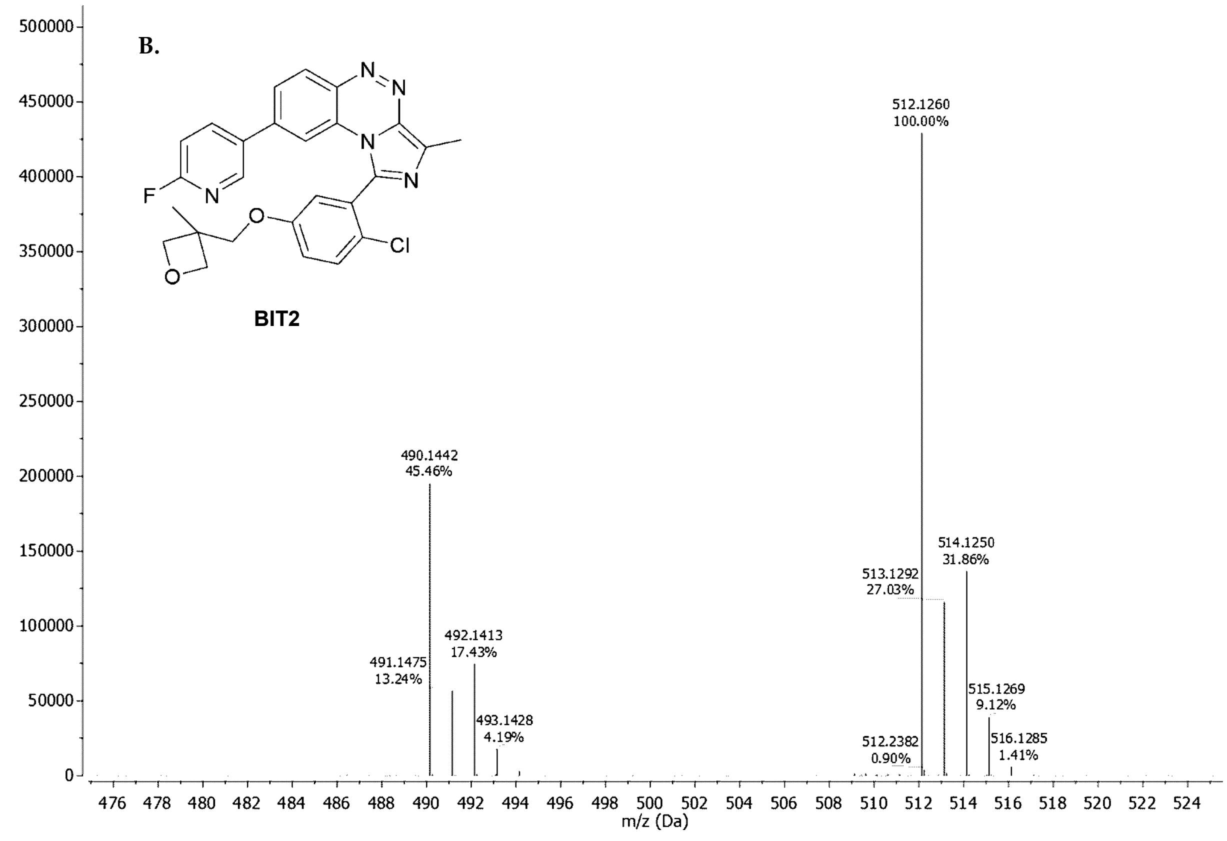

Figure S4. A. ^{19}F-NMR spectrum of **BIT2** in $CDCl_3$ and **B**. HRMS (ESI+) spectrum of **BIT2**

A.

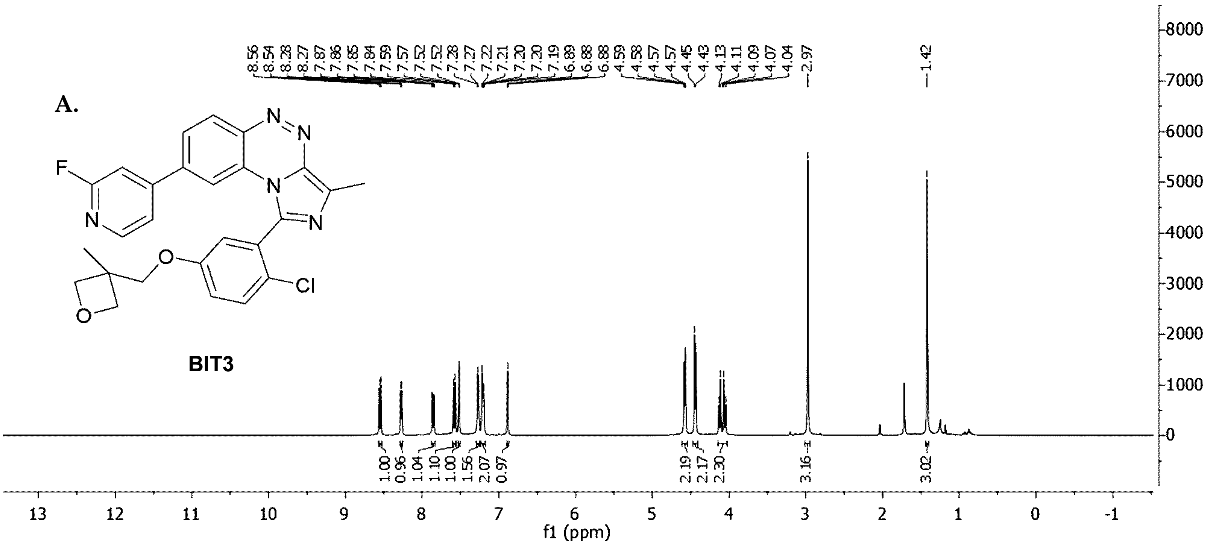

B.

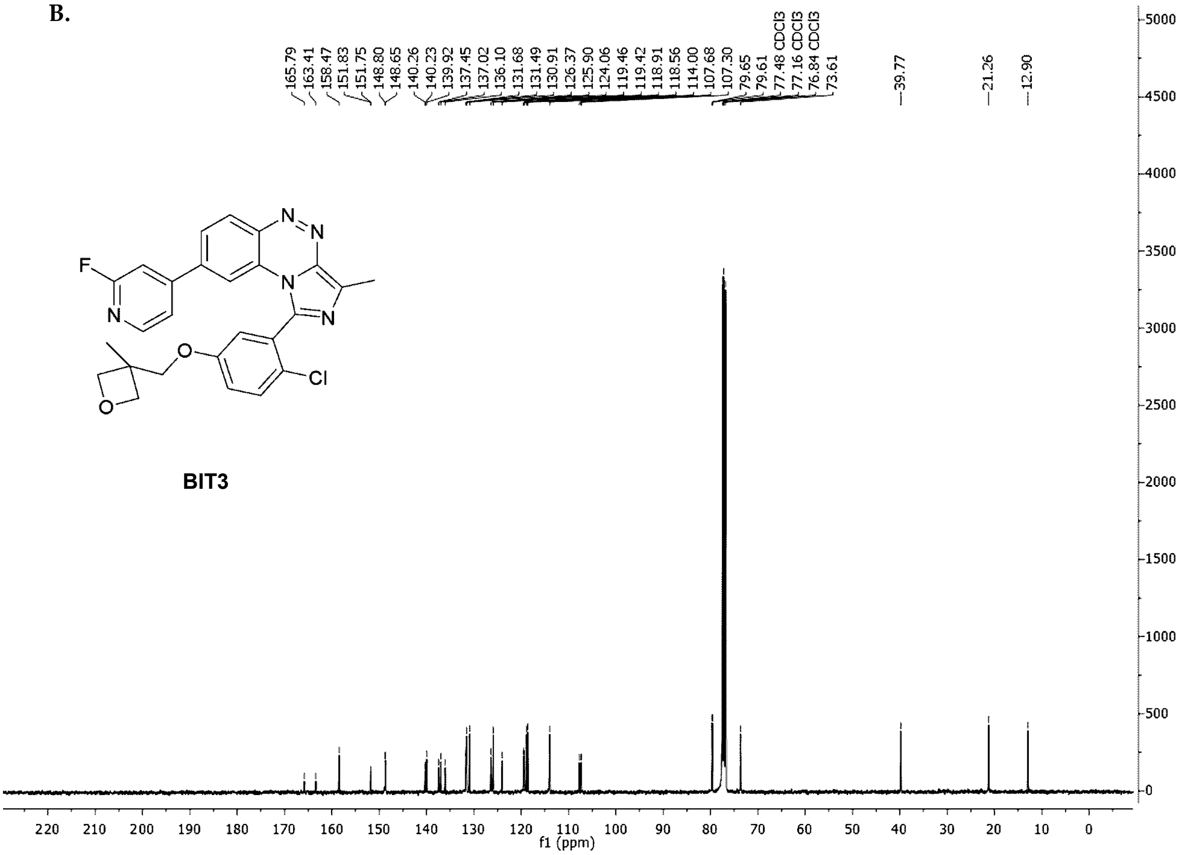

Figure S5. A. ^{1}H-NMR and **B.** ^{13}C-NMR spectrum of **BIT3** in $CDCl_3$

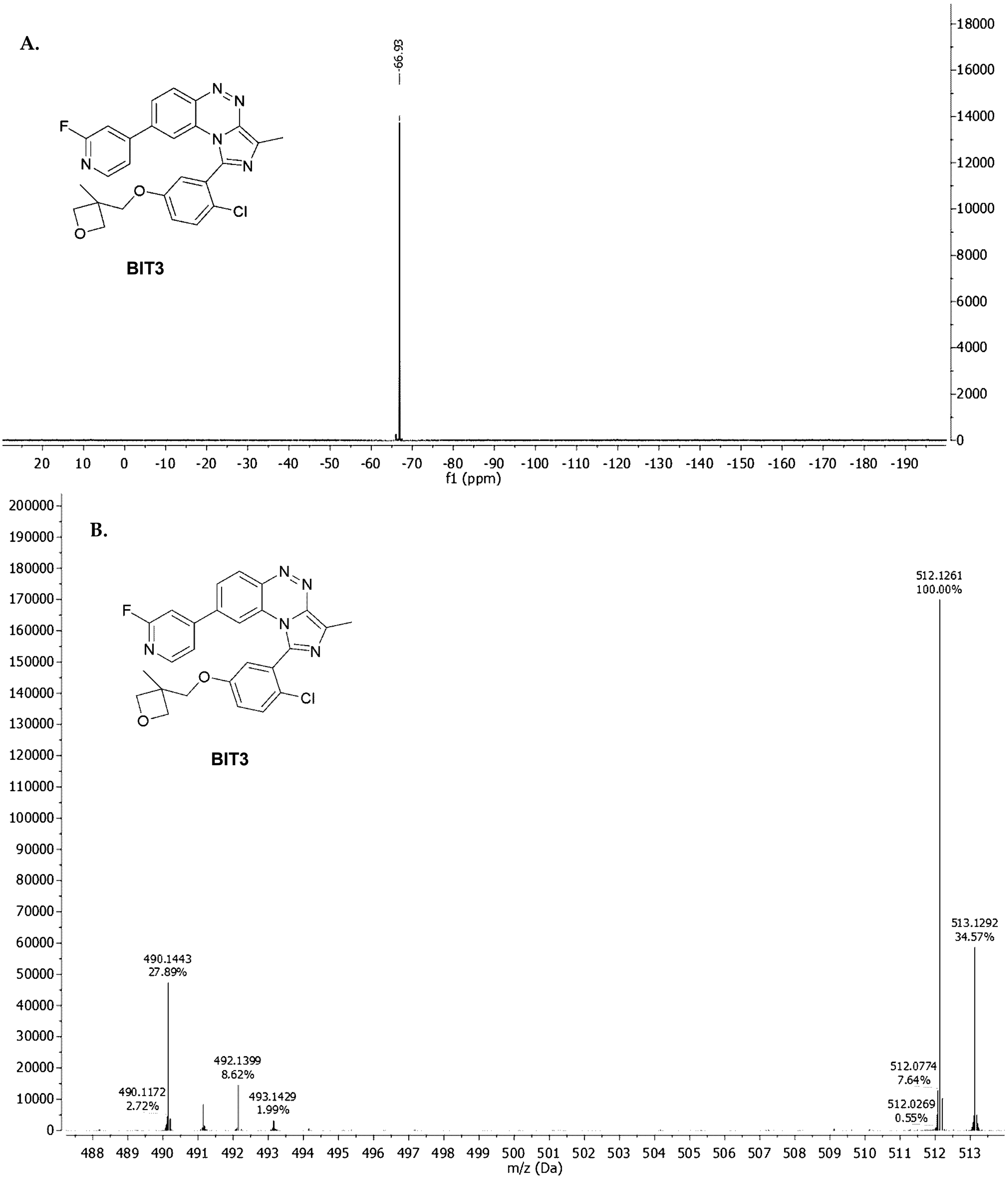

Figure S6. A. ^{19}F-NMR spectrum of **BIT3** in $CDCl_3$, **B**. HRMS (ESI+) spectrum of **BIT3**

A.

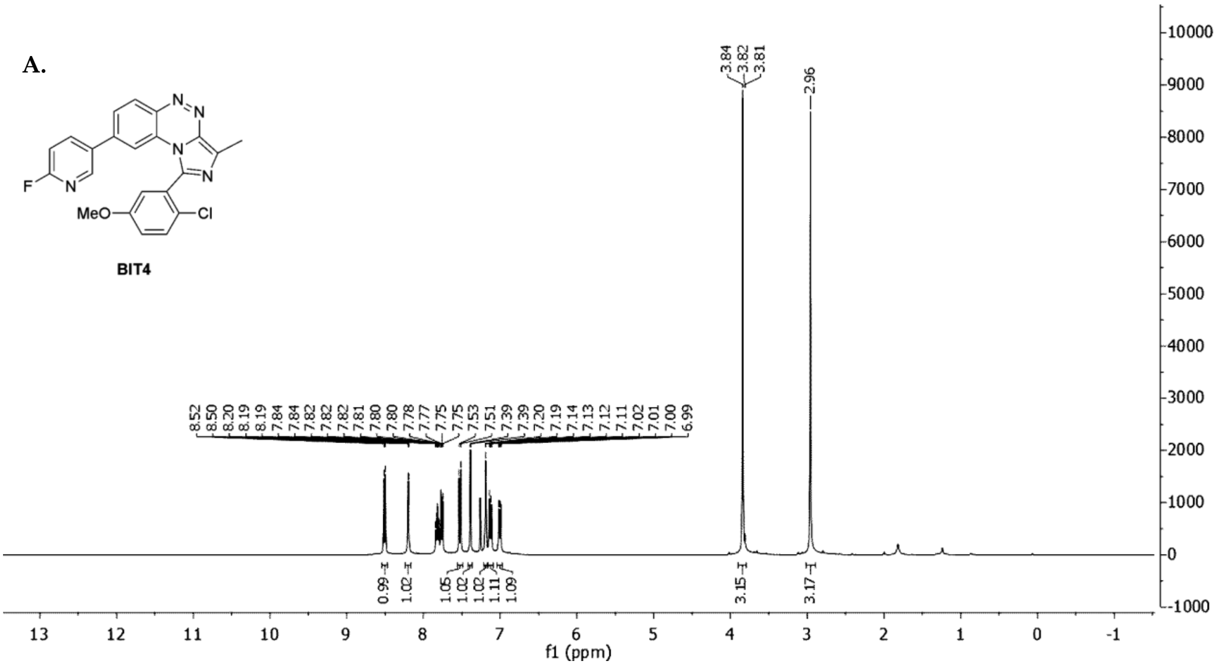

B.

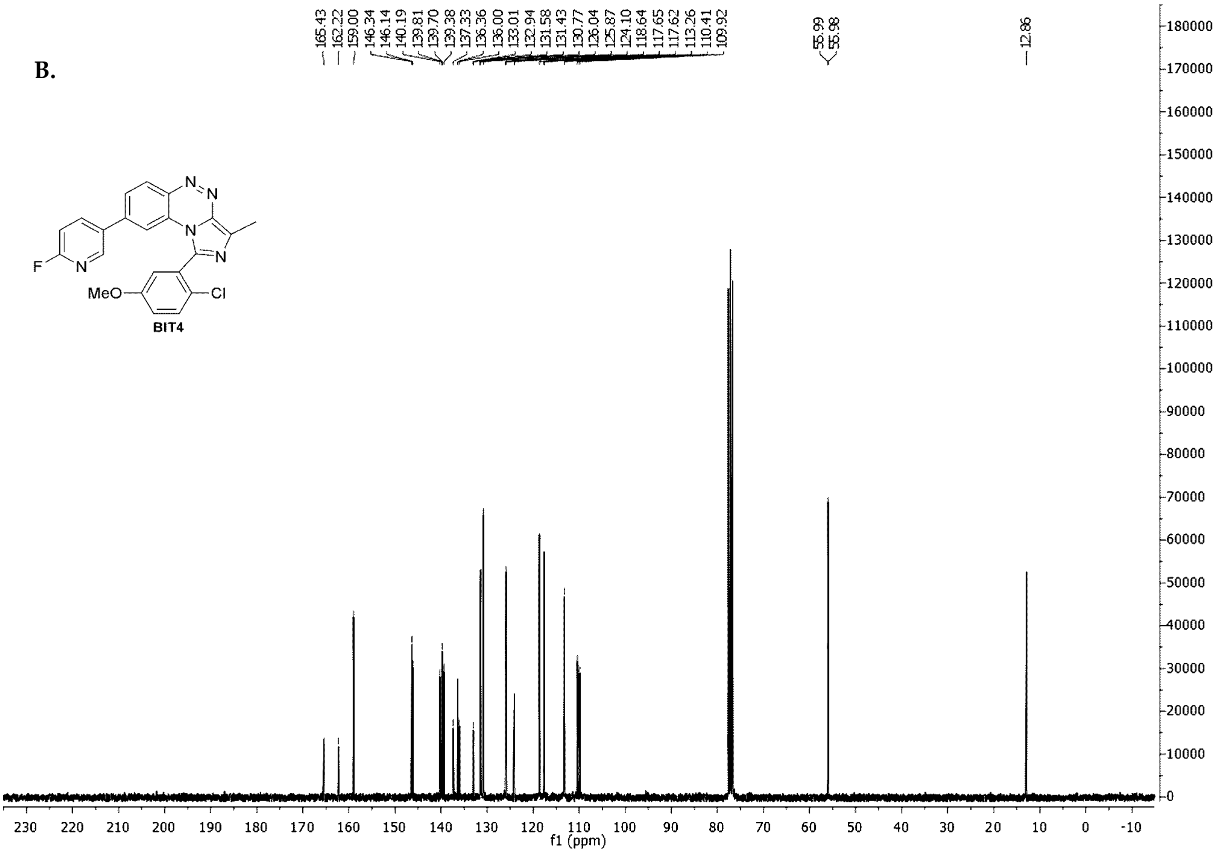

Figure S7. A. ^{1}H-NMR and **B**. ^{13}C-NMR spectrum of **BIT4** in $CDCl_3$

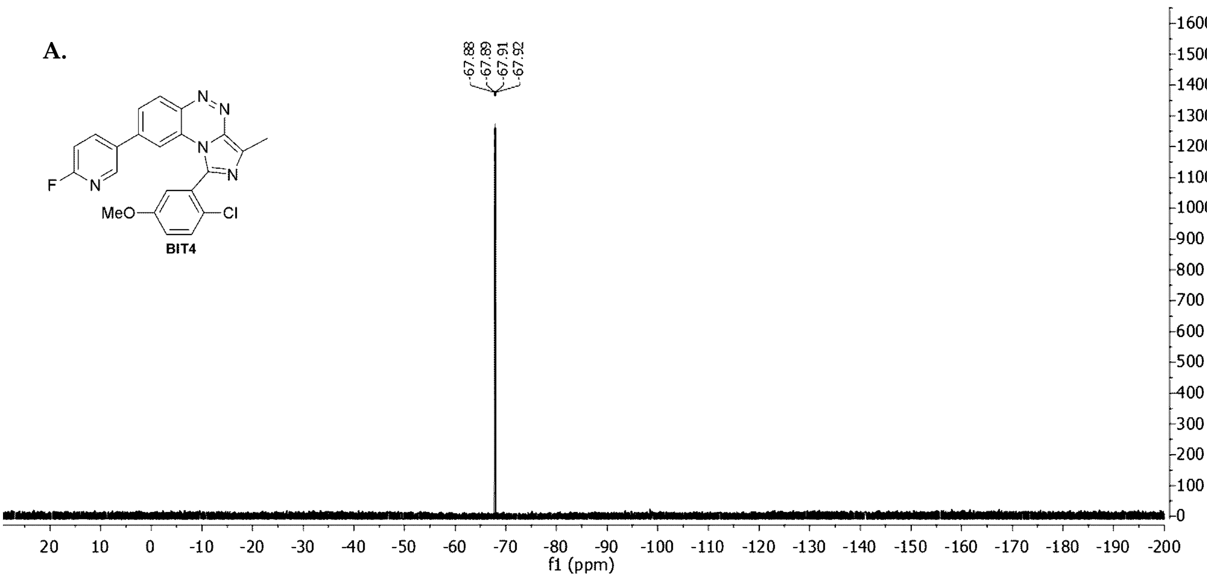

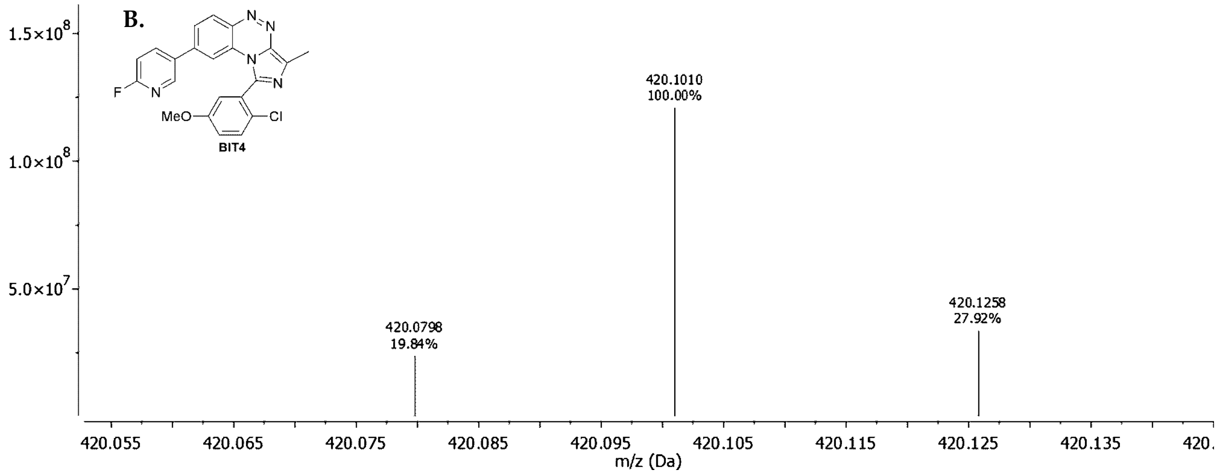

Figure S8. A. ^{19}F-NMR spectrum of **BIT4** in $CDCl_3$, **B**. HRMS (ESI+) spectrum of **BIT4**

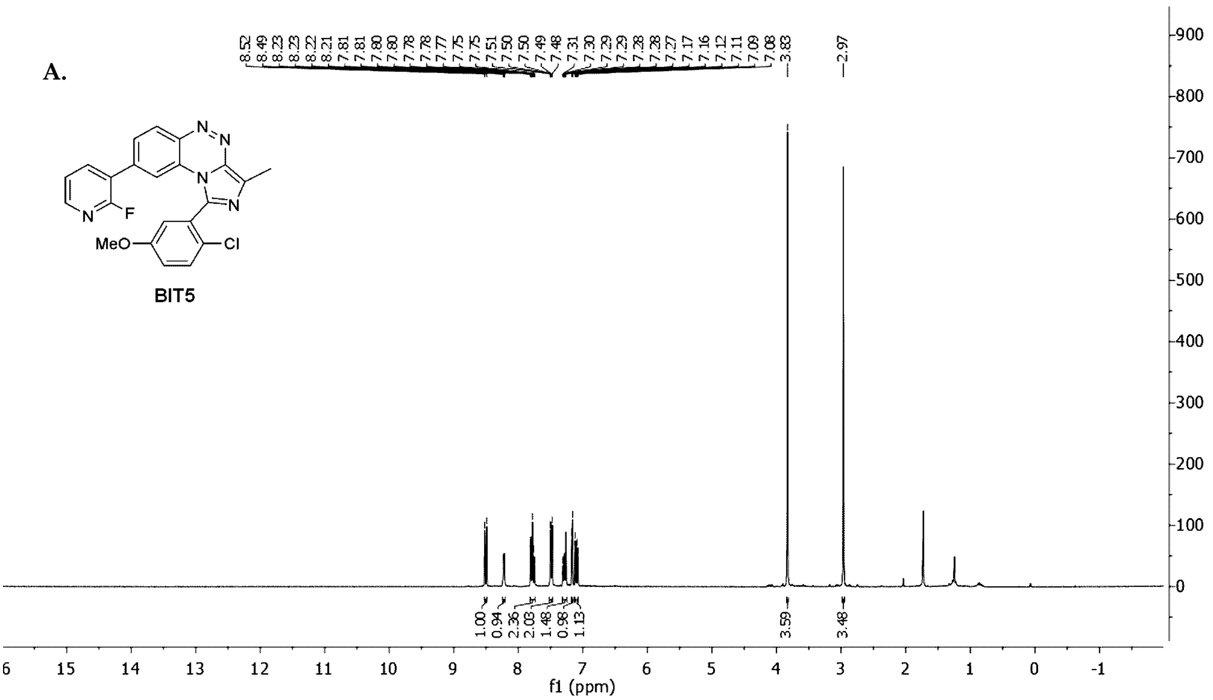

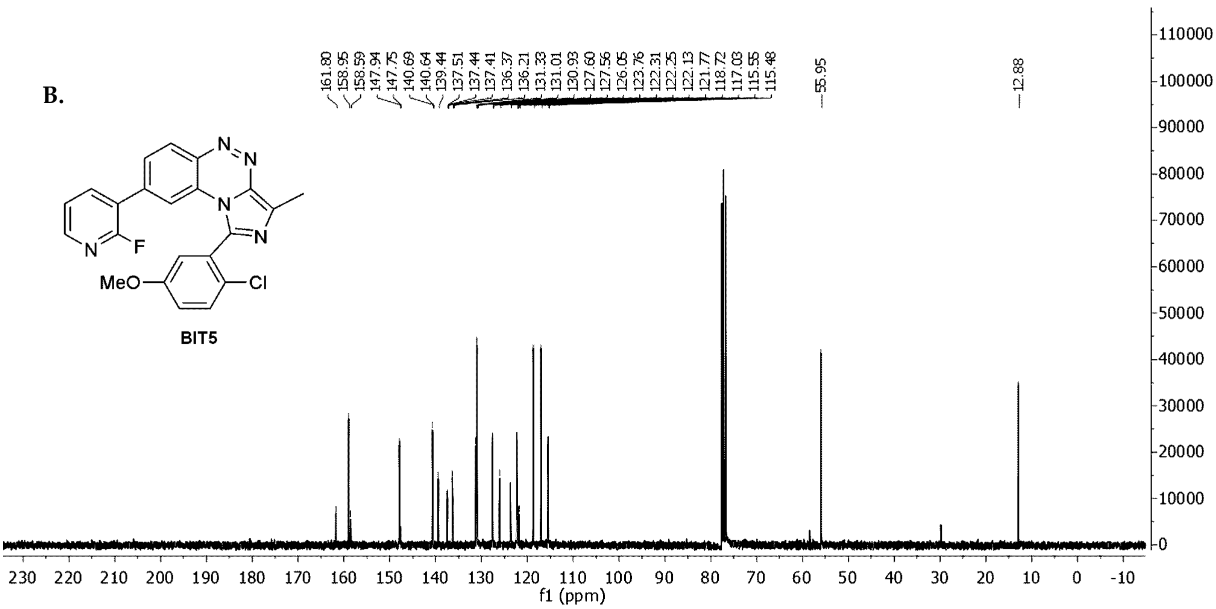

Figure S9. A. ^{1}H-NMR and **B.** ^{13}C-NMR spectrum of **BIT5** in $CDCl_3$

A.

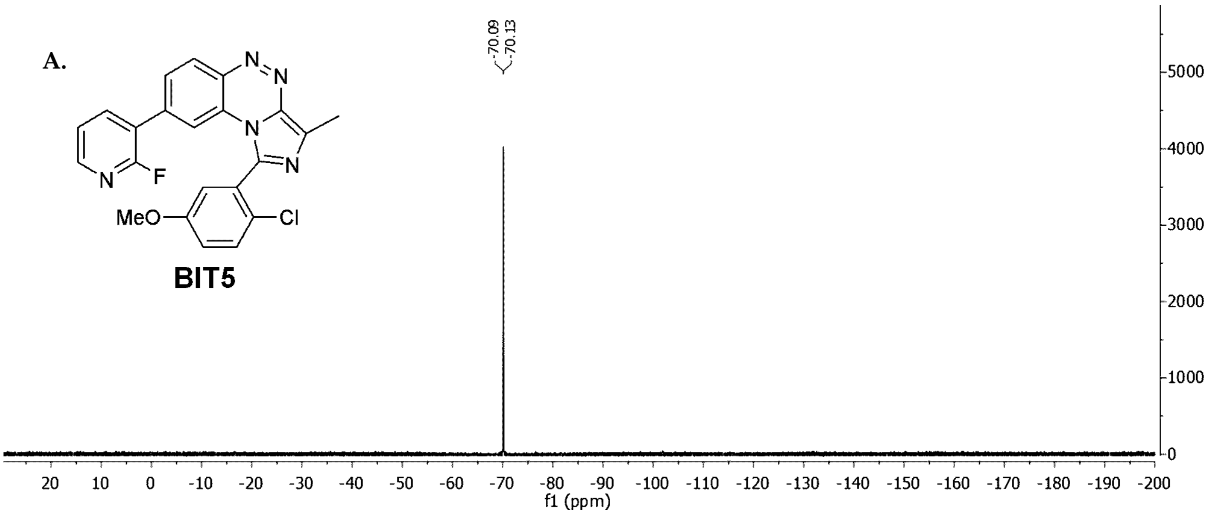

B.

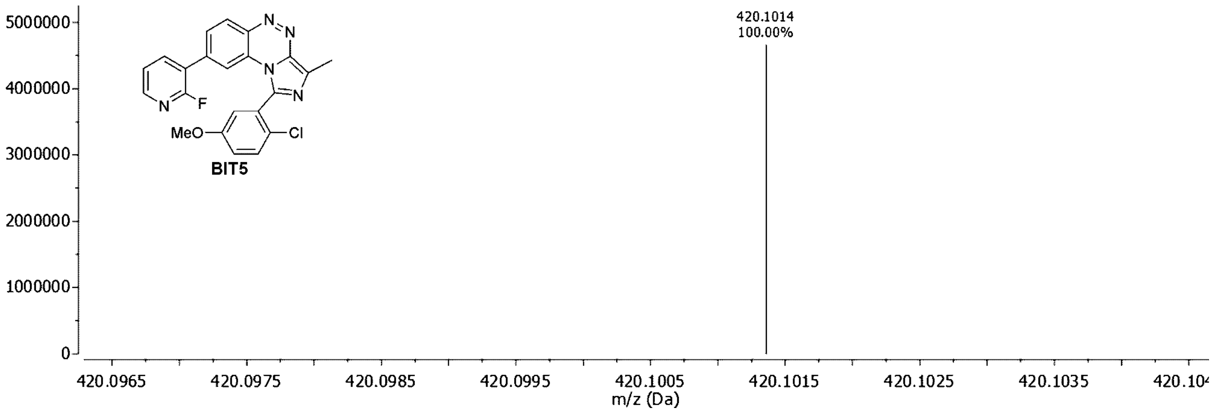

Figure S10. A. ^{19}F-NMR spectrum of **BIT5** in $CDCl_3$, **B**. HRMS (ESI+) spectrum of **BIT5**

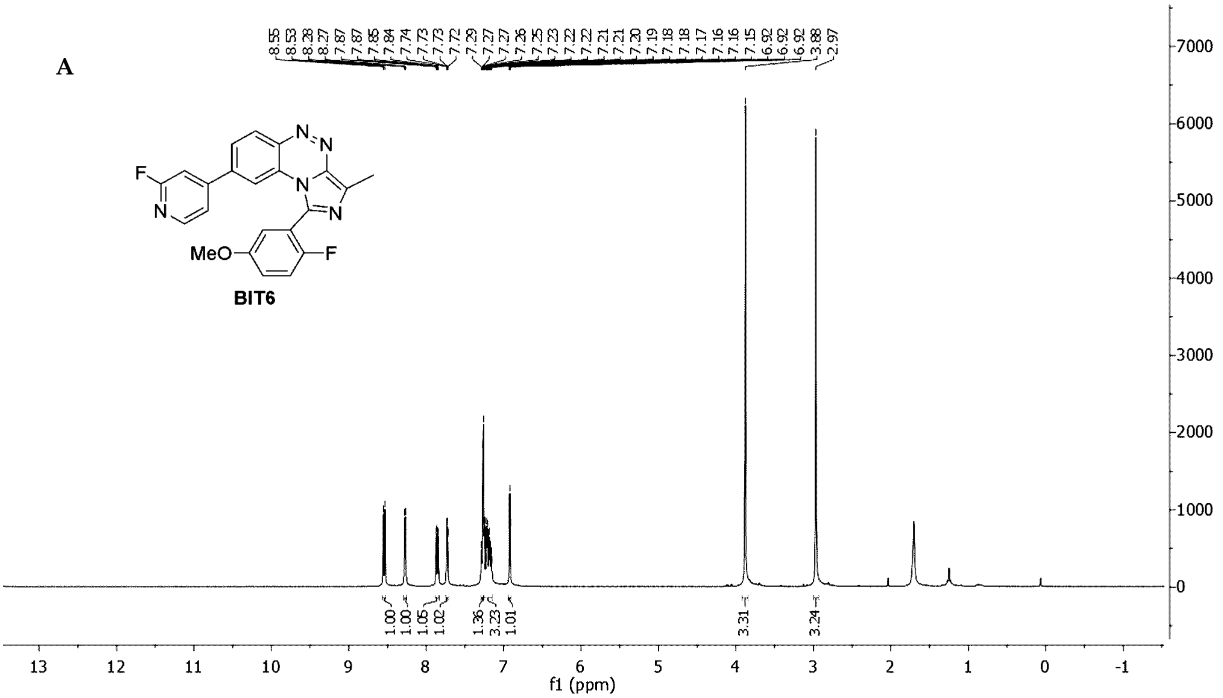

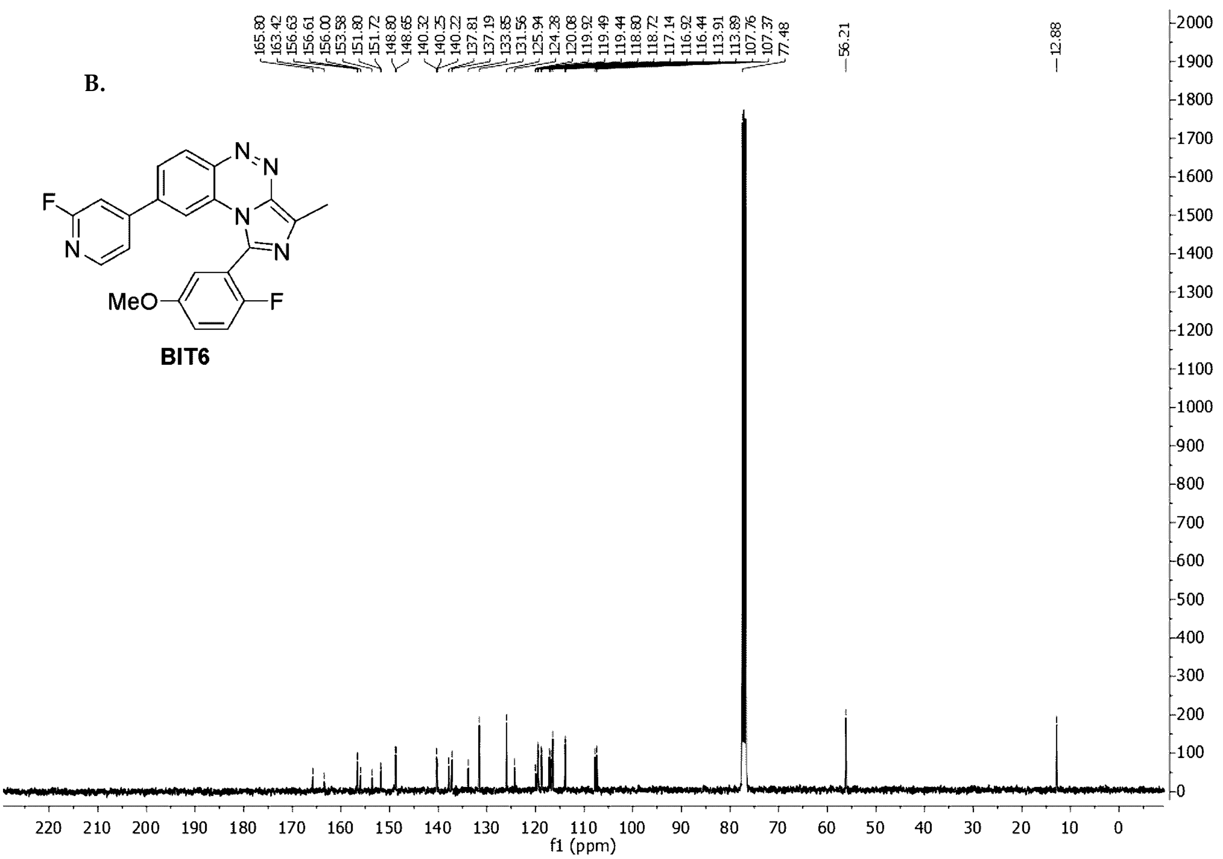

Figure S11. A. ^{1}H-NMR and **B**. ^{13}C-NMR spectrum of **BIT6** in $CDCl_3$

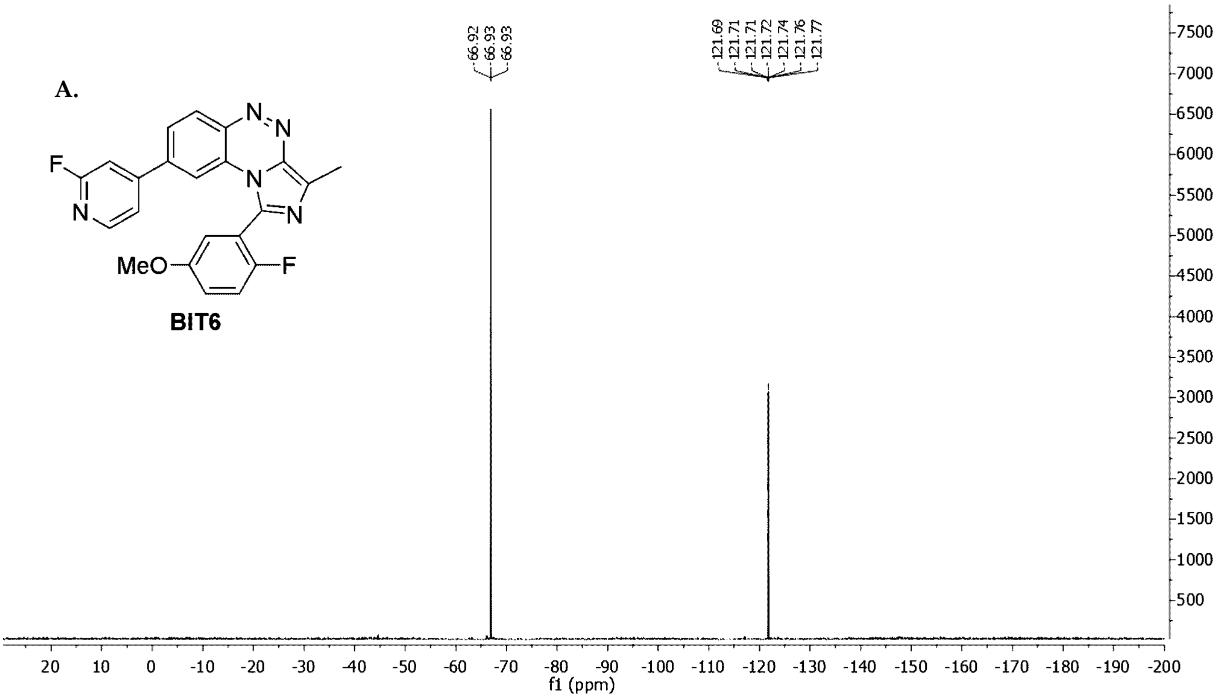

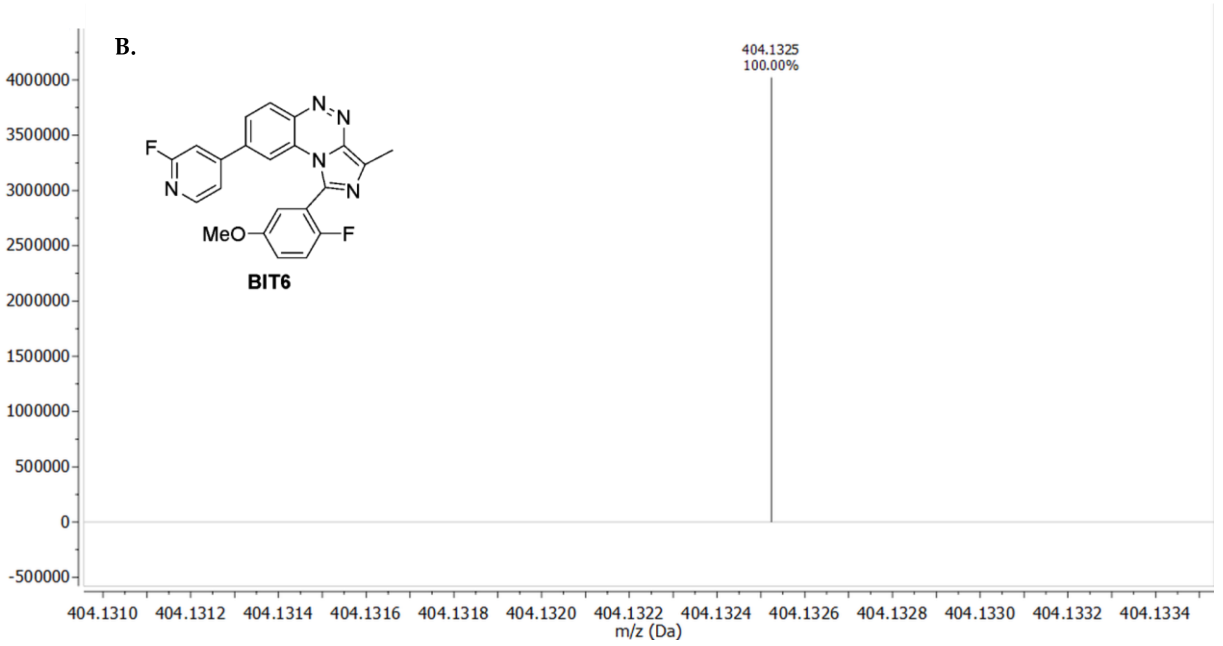

Figure S12. A. ^{19}F-NMR spectrum of **BIT6** in $CDCl_3$, **B**. HRMS (ESI+) spectrum of **BIT6**

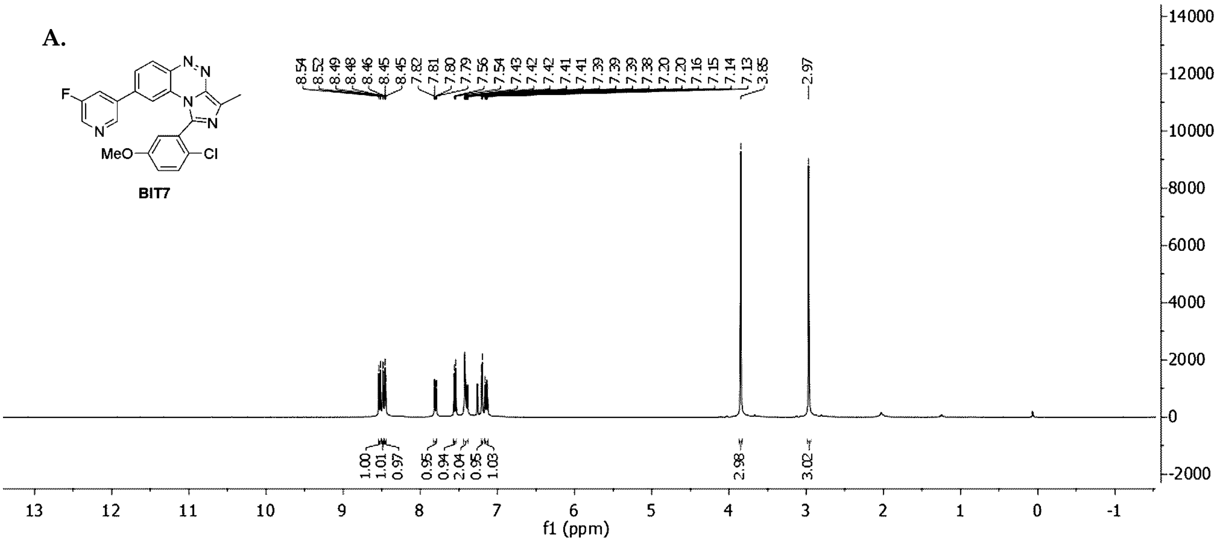

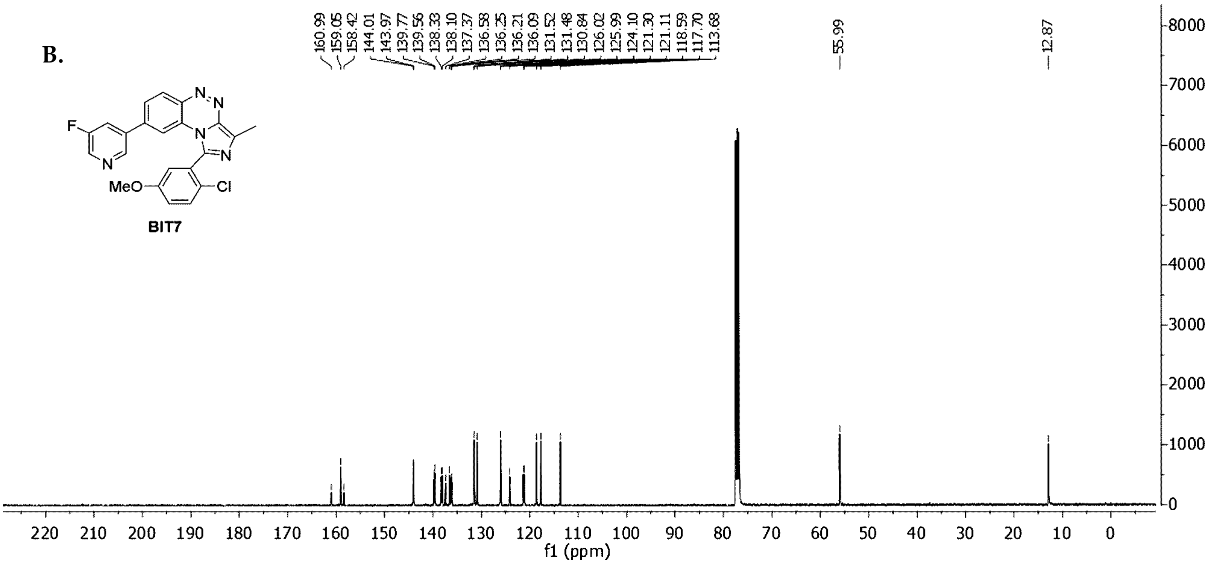

Figure S13. A. ^{1}H-NMR and **B**. ^{13}C-NMR spectrum of **BIT7** in $CDCl_3$

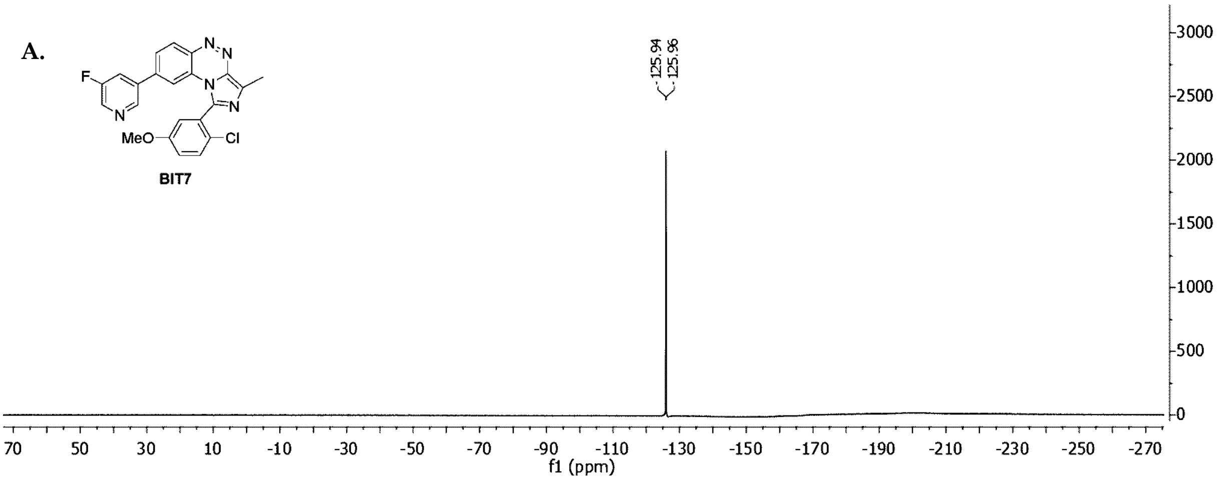

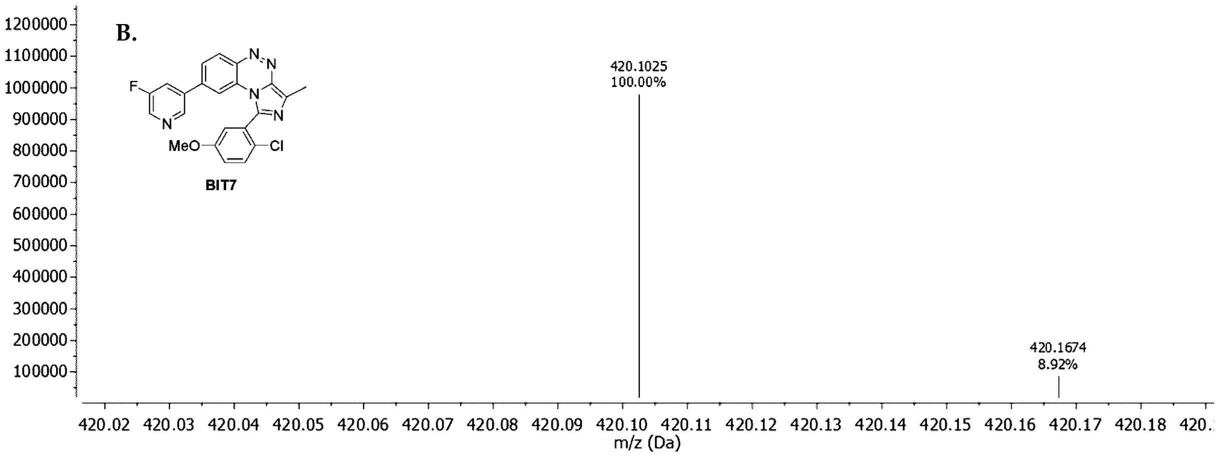

Figure S14. A. ^{19}F-NMR spectrum of **BIT7** in $CDCl_3$, **B**. HRMS (ESI+) spectrum of **BIT7**

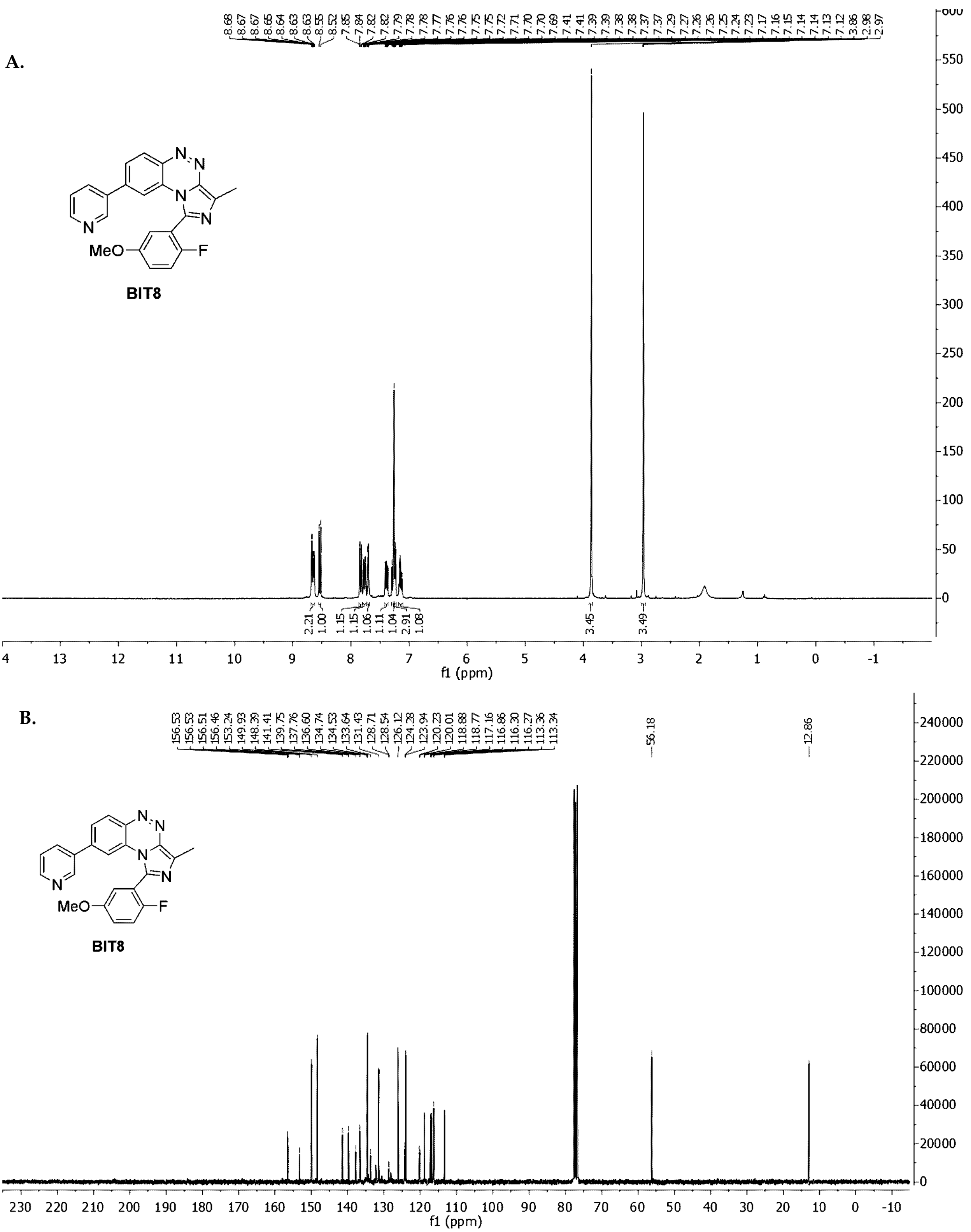

Figure S15. A. ^{1}H-NMR and **B.** ^{13}C-NMR spectrum of **BIT8** in $CDCl_3$

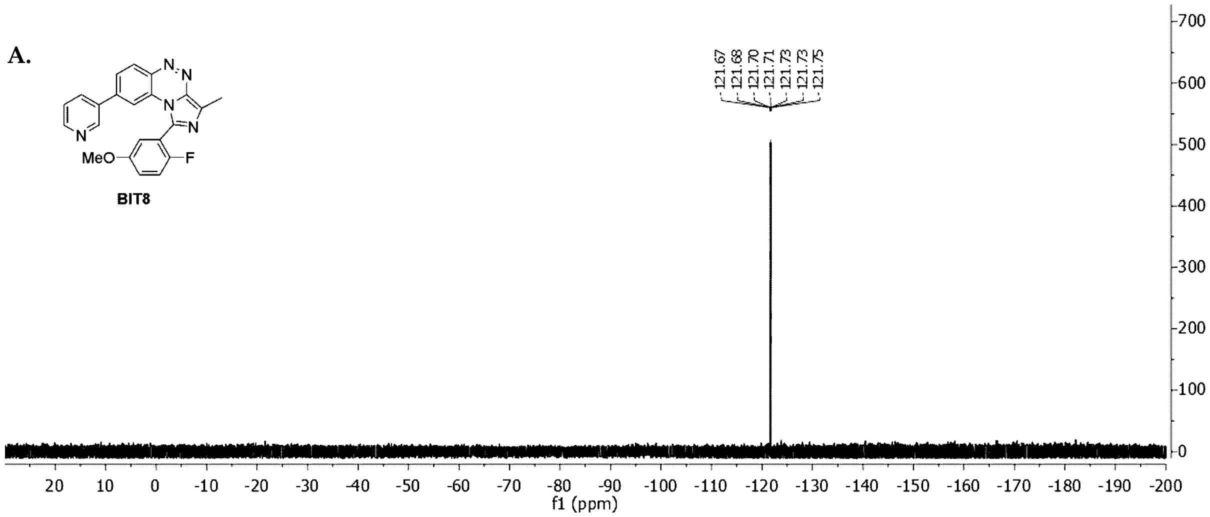

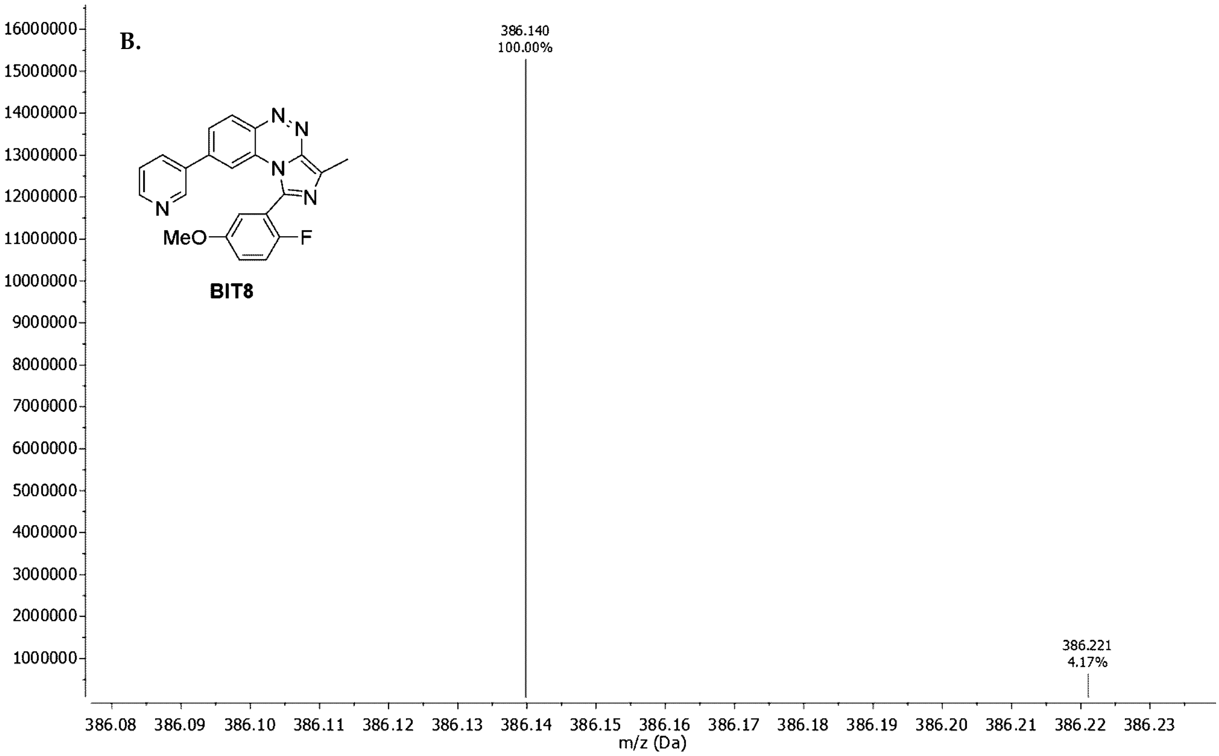

Figure S16. A. ^{19}F-NMR spectrum of **BIT8** in $CDCl_3$, **B**. HRMS (ESI+) spectrum of **BIT8**

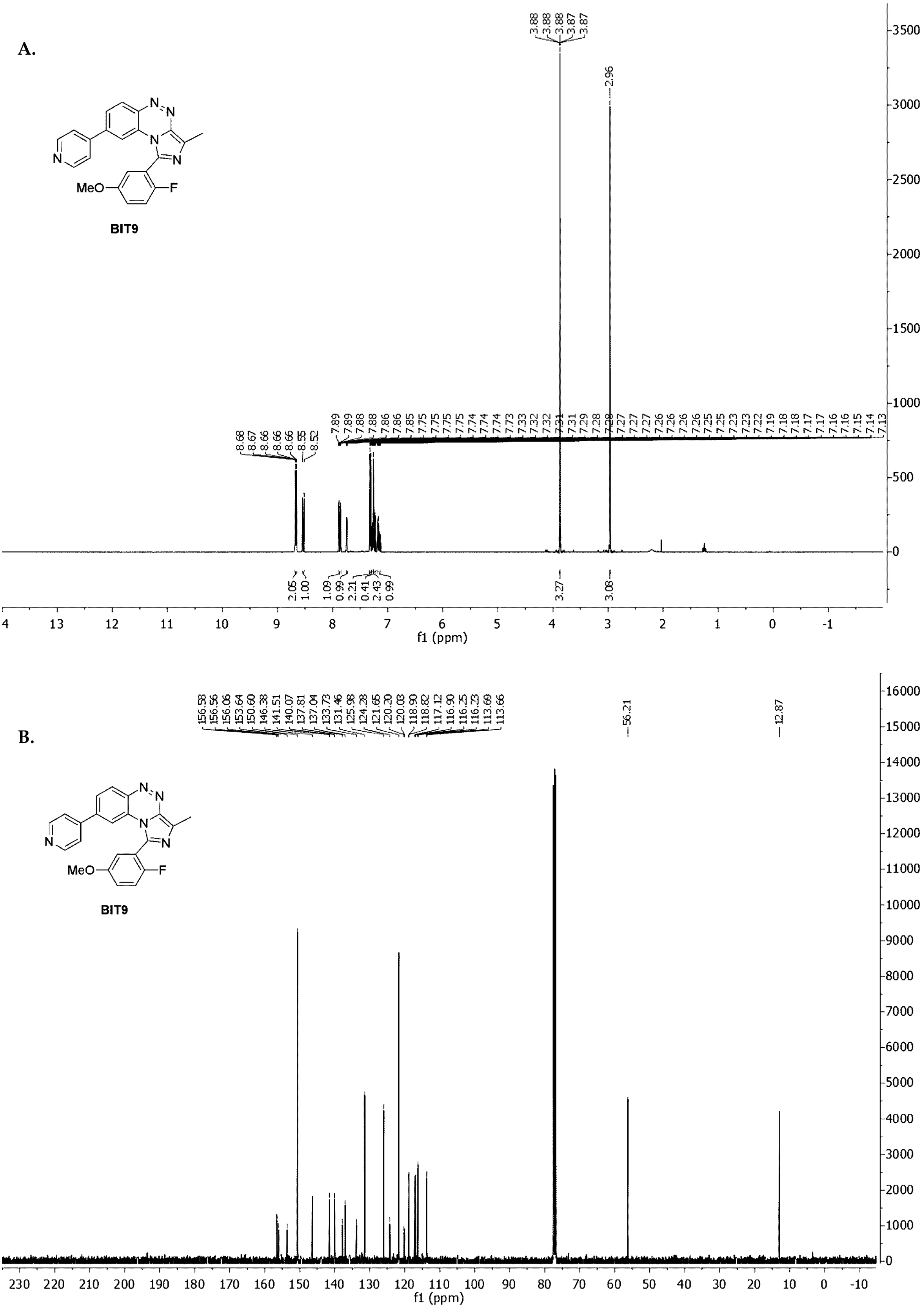

Figure S17. A. ^{1}H-NMR spectrum of **BIT9** in $CDCl_3$ and **B**. ^{13}C-NMR spectrum of **BIT9**

A.

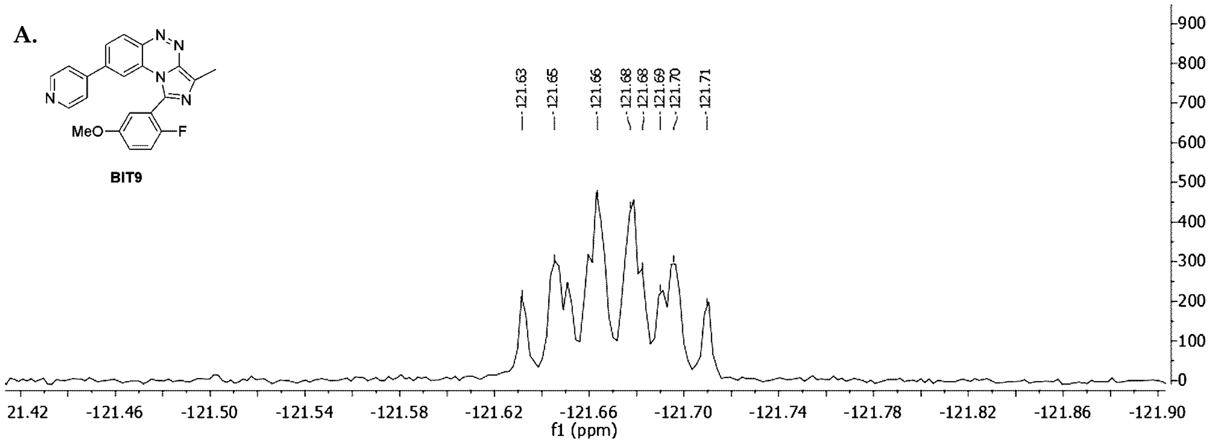

B.

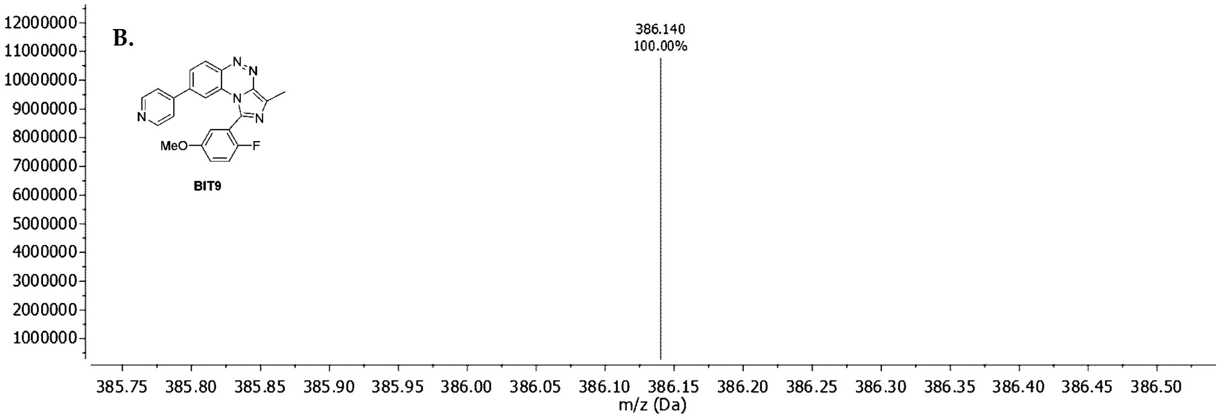

Figure S18. A. ^{19}F-NMR spectrum of **BIT9** in $CDCl_3$, **B**. HRMS (ESI+) spectrum of **BIT9**

2. In vitro evaluation (Dose Response Curve) (SB Drug Discovery-Scotland)

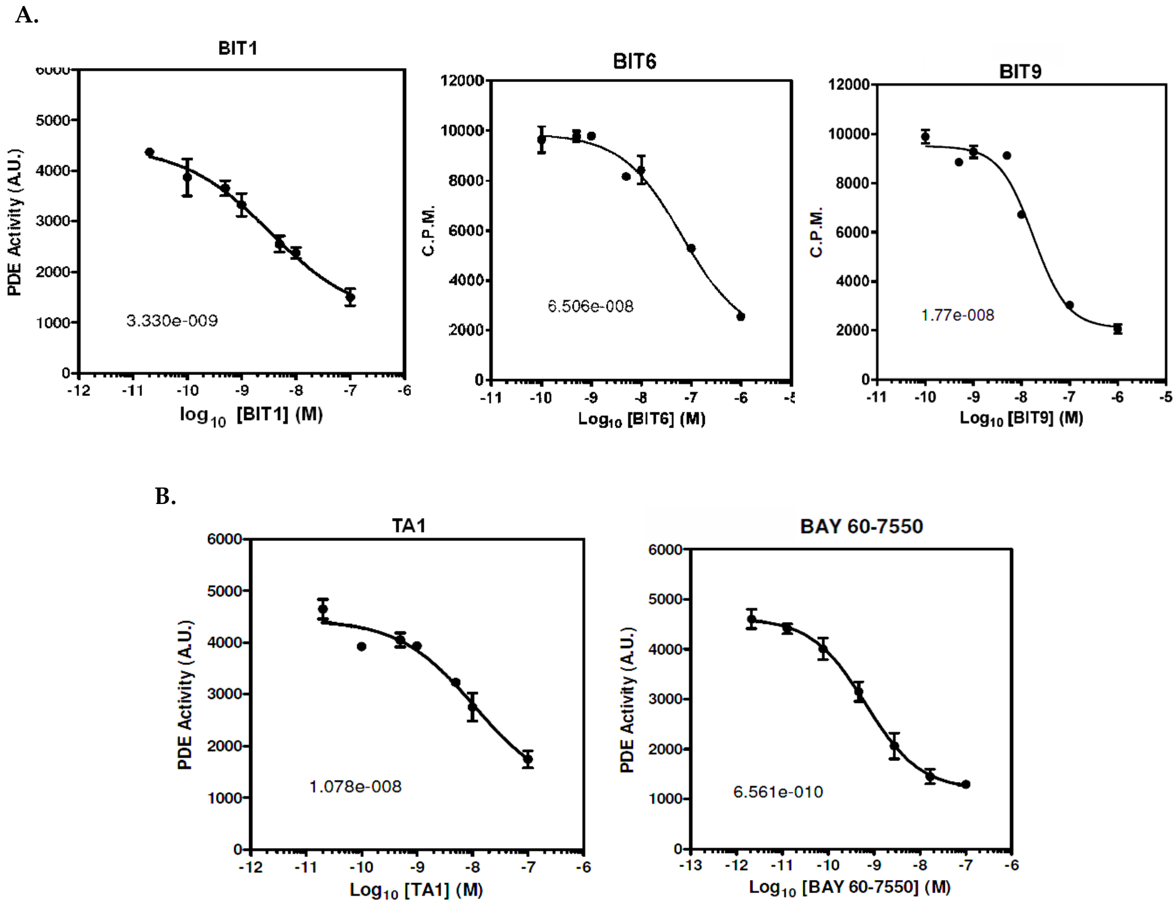

Figure S19. A. BIT1, **BIT6**, **BIT9** towards PDE2A and PDE10A, **B. BAY 60-7550** and **TA1** towards PDE2A
